# Absence of Intestinal PPARγ Aggravates Acute Infectious Colitis in Mice through a Lipocalin-2–Dependent Pathway

**DOI:** 10.1371/journal.ppat.1003887

**Published:** 2014-01-23

**Authors:** Parag Kundu, Teo Wei Ling, Agata Korecka, Yinghui Li, Rossana D'Arienzo, Ralph M. Bunte, Thorsten Berger, Velmurugesan Arulampalam, Pierre Chambon, Tak Wah Mak, Walter Wahli, Sven Pettersson

**Affiliations:** 1 Department of Microbiology, Tumor and Cell Biology (MTC), Karolinska Institutet, Stockholm, Sweden; 2 Lee Kong Chian School of Medicine, Nanyang Technological University, Singapore; 3 National Cancer Centre, Singapore; 4 Duke-NUS Graduate Medical School, Singapore; 5 Campbell Family Cancer Research Institute, Ontario Cancer Institute, University Health Network, Toronto, Ontario, Canada; 6 Institut de Génétique et de Biologie Moléculaire et Cellulaire, CNRS UMR7104, Inserm U964, Illkirch, France; 7 Center for Integrative Genomics, NCCR Frontiers in Genetics, University of Lausanne, Lausanne, Le Génopode, Switzerland; Stanford University School of Medicine, United States of America

## Abstract

To be able to colonize its host, invading *Salmonella enterica* serovar Typhimurium must disrupt and severely affect host-microbiome homeostasis. Here we report that *S.* Typhimurium induces acute infectious colitis by inhibiting peroxisome proliferator-activated receptor gamma (PPARγ) expression in intestinal epithelial cells. Interestingly, this PPARγ down-regulation by *S.* Typhimurium is independent of TLR-4 signaling but triggers a marked elevation of host innate immune response genes, including that encoding the antimicrobial peptide lipocalin-2 (Lcn2). Accumulation of Lcn2 stabilizes the metalloproteinase MMP-9 via extracellular binding, which further aggravates the colitis. Remarkably, when exposed to *S.* Typhimurium, Lcn2-null mice exhibited a drastic reduction of the colitis and remained protected even at later stages of infection. Our data suggest a mechanism in which *S.* Typhimurium hijacks the control of host immune response genes such as those encoding PPARγ and Lcn2 to acquire residence in a host, which by evolution has established a symbiotic relation with its microbiome community to prevent pathogen invasion.

## Author Summary

Enteric pathogens like *S.* Typhimurium convert the host intestine into an inflamed environment in which they are well adapted to thrive. However, the precise strategy that this pathogen employs to achieve such favorable conditions for its survival remains unclear. Here, we uncovered a novel mechanism whereby *S.* Typhimurium inhibits the expression of the transcription factor PPARγ in the host intestine, surprisingly without TLR-4 involvement; this inhibition worsened the severity of the host's colitis. Subsequent detailed analysis revealed that colitis severity was coupled with elevated levels of antimicrobials like Lcn2, which stabilized the pro-inflammatory endopeptidase MMP-9 in the intestinal milieu. Combination of this escalated antimicrobial action together with enhanced protease activity disrupted the intestinal homeostasis, promoting an inflamed environment suitable for *S.* Typhimurium. Interestingly, using Lcn2 mutant mice we show that lack of Lcn2 effectively reduced tissue damage and the degree of inflammation, thus supporting a pivotal role of Lcn2 and MMP-9 in infectious colitis. Our data suggests a model whereby the pathogenesis of *S.* Typhimurium involves manipulation of the host innate immune and protease system, here illustrated by PPARγ, Lcn2 and MMP-9, to establish colonization and infection within the host.

## Introduction

*Salmonella enterica* serovar Typhimurium is a Gram-negative, facultative intracellular pathogen that causes a wide array of disorders ranging from systemic disease to enterocolitis in multiple hosts [1]. In mice, *S.* Typhimurium normally causes a disease that resembles systemic typhoid fever. However, compromising the gut microbiome with antibiotics prior to *S.* Typhimurium infection in mice has been used to mimic salmonellosis in humans, which involves increased *S.* Typhimurium colonization of the intestine coupled with a marked host-induced inflammatory response leading to colitis [2]. Recent reports indicate that this massive inflammatory response elicited by *S.* Typhimurium is associated with increased secretion of the interleukins IL-17 and IL-22 [3], [4], which are critical components of mucosal immunity to bacterial pathogens in the gut. In particular, the IL-17/IL-22 axis mediates the recruitment of antimicrobial peptides from the intestinal epithelial compartment, including lipocalin-2 (Lcn2) [3]–[7]; these peptides dramatically affect the gut microbiota.

Lcn2 (also known as SIP24, 24p3, NGAL, uterocalin, and siderocalin) was first co-purified and found to be covalently associated with human neutrophil gelatinase (matrix metalloproteinase (MMP)-9) [8], [9]. This association between Lcn2 and MMP-9 has been shown to protect MMP-9 from degradation and to preserve its enzymatic activity [8], [10]. In addition, Lcn2 functions in mammalian innate immunity by chelating bacterial siderophores, thereby sequestering iron from bacteria and inhibiting their growth [11], [12]. Intriguingly, *S.* Typhimurium appears to be resistant to Lcn2, since its population in the intestinal milieu expands dramatically during inflammation [4], [13], [14]. This unique strategy is achieved by genes such as those in the *iroN iroBCDE* gene cluster, which encodes salmochalin, a siderophore that does not bind Lcn2 [4], [13], [15], [16], thus conferring a competitive advantage to *S.* Typhimurium over other microbes during growth in the inflamed gut.

Recently, peroxisome proliferator-activated receptor gamma (PPARγ) has been shown to be regulated by a number of bacterial pathogens including *Helicobacter pylori* and *Mycobacterium tuberculosis* [17]–[19], greatly impacting disease severity. PPARγ is a member of the nuclear receptor superfamily of ligand-dependent transcription factors and is predominantly expressed in adipose tissue and colonic epithelium [20], [21]. Expression has also been detected in colonic macrophages and in T and B cells of humans and rodents [20], [22]. PPARγ has been proclaimed to be a master regulator of inflammation, a role that is achieved in part by antagonizing the activities of the transcription factors AP-1, STAT, and NFκB [23], [24]. *In vivo* studies have demonstrated that PPARγ ligands actively suppress the inflammatory response by attenuating the production of chemokines and cytokines secreted from epithelial cells, macrophages, and T and B lymphocytes [23]–[26].

The role of PPARγ in the etiology and treatment of colitis has been of great interest, because its ligands have long been used to treat type-2 diabetes and are known to decrease the severity of colitis induced in mouse models [27]–[33]. Moreover, PPARγ^+/−^ heterozygous mice exhibit increased susceptibility to experimentally induced colitis, indicating PPARγ's involvement in maintaining gut homeostasis [28], [33]. Furthermore, the observation that intestinal epithelium-specific ablation of PPARγ aggravates dextran sodium sulfate (DSS)-induced colitis demonstrates the strong influence of intestine-derived PPARγ on colitis severity [20]. Studies in human subjects have revealed that colonic epithelial cells from ulcerative colitis patients display drastically reduced expression of PPARγ, suggesting that its presence in gut epithelium may have a protective effect against colonic inflammation in humans [29]. Despite these observations, the role of PPARγ in *S.* Typhimurium-induced infectious colitis remains unknown.

In this study, we explored whether *S.* Typhimurium regulates host PPARγ levels during infectious colitis and evaluated PPARγ's contributions to the etiology of the disease. Our data reveal that *S.* Typhimurium inhibits PPARγ expression in the intestinal epithelium, which triggers a massive innate immune response that includes expression of Lcn2. Selective epithelial ablation of PPARγ dramatically increased Lcn2 expression and its secretion after *S.* Typhimurium challenge, confirming the importance of epithelium-derived PPARγ in colitis. Furthermore, increased secretion of Lcn2 stabilized MMP-9 via direct protein-protein interaction, which further aggravated the colitis. Finally, we demonstrate that Lcn2-null mice exposed to *S.* Typhimurium displayed significantly less-severe colitis.

## Results

### Regulation of PPARγ in the mouse colon by *S.* Typhimurium

**Figure 1:**
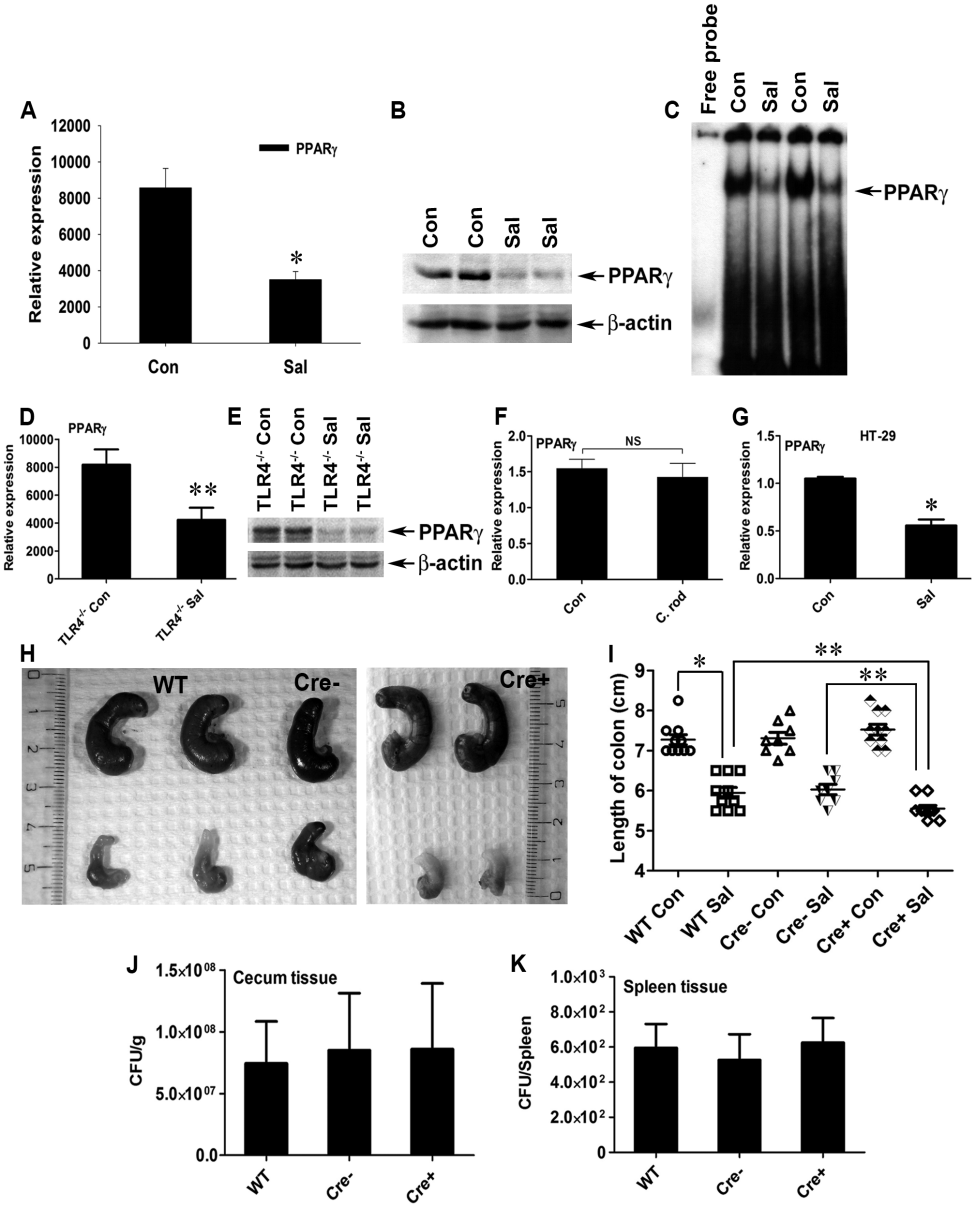
*S.* Typhimurium down-regulates PPARγ while inducing colitis in C57BL/6 mice. (A, B, and C) Groups of 8–10-week-old streptomycin-pretreated C57BL/6 mice were mock- (Con) or *S.* Typhimurium-infected (Sal) and sacrificed after 24 h (10 mice per group). PPARγ expression in colonic scrapings was analyzed by real-time PCR (A) and by immunoblotting (B). (C) Electromobility shift assay of PPARγ activity in the nuclear extracts of colonic scrapings. (D and E) Age-matched, streptomycin-pretreated TLR4^−/−^ mice were mock- or *S.* Typhimurium-infected and sacrificed after 24 h (5 mice per group). Colonic expression of PPARγ was analyzed by real-time PCR (D) or immunoblotting (E). (F) Metronidazole-pretreated C57BL/6 mice were mock- or *Citrobacter rodentium*-infected, sacrificed 6 days after infection, and PPARγ expression in the colon was analyzed by real-time PCR. (G) HT-29 cells were mock- or *S.* Typhimurium-infected for 6 h, incubated for another 18 h without the pathogen, and PPARγ expression was analyzed by real-time PCR. (H) Macroscopic image of whole cecum after mock or *S.* Typhimurium infection in C57BL/6 (WT), PPARγVillinCre− (Cre−), or PPARγVillinCre+ (Cre+) mice. (I) Quantitation of colon lengths in the respective mouse groups. Recovery of *S.* Typhimurium from cecum tissue (J) and spleen (K) 24 h after infection. Error bars depict ± standard error of the mean. *p<0.001, **p<0.05. CFU, colony-forming units; NS, not significant.

Although PPARγ signaling controls various cellular processes during inflammation and pathogenesis, its regulation during *S.* Typhimurium-induced colitis remains unexplored. To gain insight into PPARγ's role in infectious colitis, streptomycin-pretreated C57BL/6 mice were infected with *S.* Typhimurium. An incubation period of 24 h was deliberately selected to evaluate the early phase of PPARγ response, which is crucial for further downstream effector regulation. Strikingly, *S.* Typhimurium infection resulted in ∼60% down-regulation of PPARγ gene expression in the colon, as detected by real-time PCR (Fig. 1A). Immunoblotting revealed a similar down-regulation of PPARγ expression at the protein level (Fig. 1B) in the colon 24 h after infection. As expected, this down-regulation resulted in a marked reduction in the DNA-binding activity of PPARγ in colonic cells *in vivo* (Fig. 1C), thus reducing PPARγ's tight control over its potential targets. A similar effect was detected in the cecum of infected mice (Fig. S1A–C).

Since a previous study reported Toll-like receptor (TLR)-4-dependent PPARγ regulation by microbial lipopolysaccharide in macrophages [34], we asked whether this negative regulation of PPARγ by *S.* Typhimurium was TLR-dependent. A significant increase in the expression of gene encoding TLR-4, but not TLR-2 and TLR-5, was observed in the infected mice, suggesting that TLR4 may be involved in regulating PPARγ (Fig. S2A–C). However, *S.* Typhimurium infection in TLR-4^−/−^ mice resulted in a similar decline in PPARγ expression (Fig. 1D and E), suggesting that this PPARγ regulation was independent of TLR-4. However, the expression of TLR-2 and TLR-5 gene in the infected TLR-4^−/−^ mice remained unchanged, negating the possibility of their increased activity in the absence of TLR-4 (Fig. S2D and E). To confirm the specificity of PPARγ deregulation by *S.* Typhimurium, another potential gut pathogen, *Citrobacter rodentium*, was used to infect mice. *C. rodentium* was unable to alter host PPARγ levels, confirming that the process of PPARγ down-regulation by *S.* Typhimurium was not a general effect (Fig. 1F).

Next, we set out to identify the cell types that predominantly respond to *S.* Typhimurium. A sharp decrease in the gene expression of PPARγ occurred when cultures of colonic epithelial cells (HT-29 cells) were infected with *S.* Typhimurium, suggesting the importance of the intestinal epithelium in this process (Fig. 1G). These observations not only indicate that *S.* Typhimurium infection directly impacts PPARγ levels in the colonic epithelium, but also suggest that PPARγ's pivotal role in homeostasis within the intestinal tract affects infectious colitis.

### Specific ablation of PPARγ in the intestinal epithelium aggravates *S.* Typhimurium-induced colitis

To better understand the implications of this PPARγ regulation by *S.* Typhimurium, we bred mice harboring a floxed *Pparγ* (PPAR*γ* ^fl/fl^) to mice expressing the *Cre* transgene under control of the promoter of the *villin* gene. These mice, in which cre recombinase mediated the targeted disruption of *PPARγ* in intestinal epithelial cells, were designated PPARγVillinCre+ mice and were used in parallel with littermate control PPARγVillinCre- or wild-type (C57BL/6) mice. Interestingly, *S.* Typhimurium infection resulted in more severe colitis in the PPARγVillinCre+ mice compared to PPARγVillinCre- or wild-type mice at 24 h (Fig. 1H and I). Shortening and thickening of the cecum and the colon, which are hallmarks of colitis, were much more pronounced in PPARγVillinCre+ mice than in wild-type or PPARγVillinCre- mice (Fig. 1I). Of note, the *S.* Typhimurium-infected TLR-4^−/−^ mice and the wild-type (C57BL/6) mice infected with *C. rodentium* exhibited significantly shortened colons, indicating active colitis (Fig. S2F and G).

**Figure 2:**
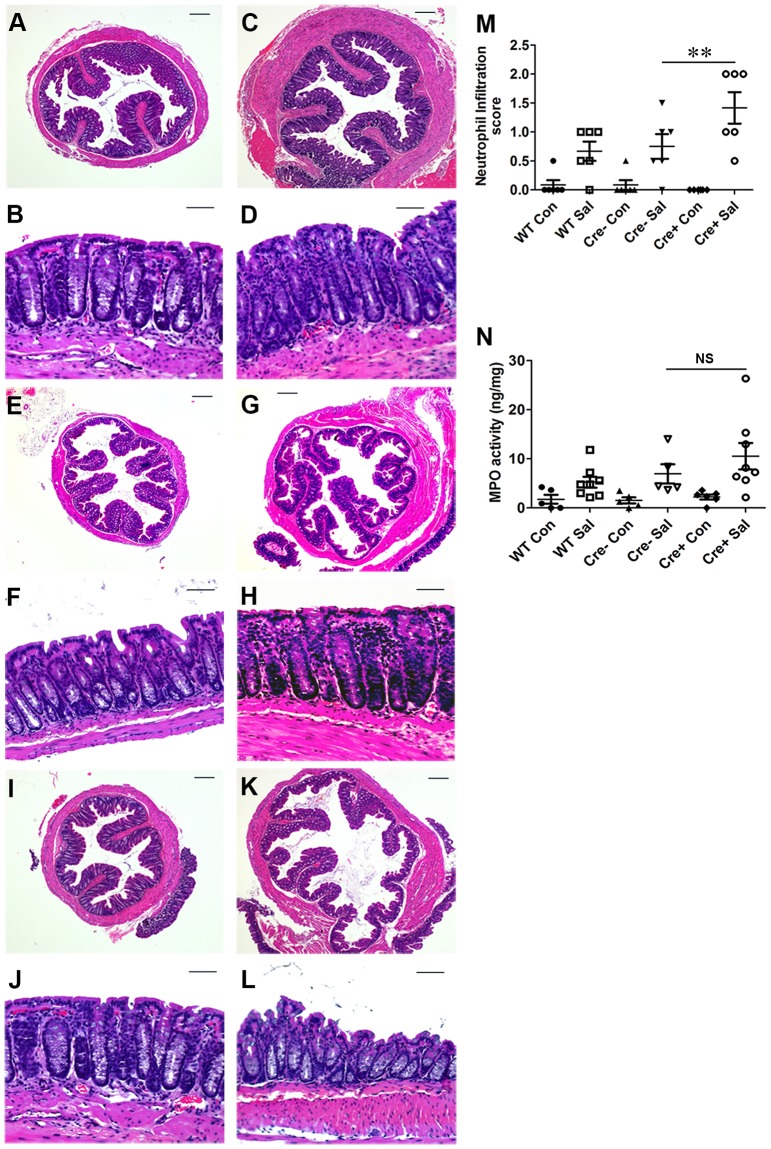
Colitis severity is more pronounced in PPARγVillinCre^+^ mice than in wild-type mice. Sections of colon from mock- or S. Typhimurium-infected wild-type (C57BL/6) and PPARγVillinCre^+^ mice were stained with hematoxylin and eosin (6 mice per group). (A and B) Wild-type mice mock-infected. (C and D) Wild-type mice infected with S. Typhimurium. PPARγVillinCre− mice mock-infected (E and F), or infected with S. Typhimurium (G and H). PPARγVillinCre^+^ mice mock-infected (I and J), or infected with S. Typhimurium (K and L). All scale bars are 100 µm. Pathology scoring was performed for neutrophil infiltration (M). (N) Myeloperoxidase (MPO) activity in colonic extracts of mice, measured per mg of total protein. In panels M and N, mock (Con)- or S. Typhimurium (Sal)-infected PPARγVillinCre^+^ (Cre^+^) or littermate control PPARγVillinCre− (Cre−) mice were examined. Error bars  = ±standard error of the mean. **p<0.01.

*S.* Typhimurium cells were recovered in similar numbers from cecum tissue and from the spleens of wild-type, PPARγVillinCre-, and PPARγVillinCre+ mice 24 h after infection (Fig. 1J and K), indicating that infection in the cecum as well as systemic dissemination of *S.* Typhimurium were comparable between these groups. Histological analysis further revealed that apart from increased infiltration of inflammatory cells, tissue damage was more in the colons of PPARγVillinCre+ mice compared to PPARγVillinCre- mice and to wild-type mice after infection (Fig. 2). Notably, similar levels of the epithelial cell markers villin 1, cytokeratin 8, and cytokeratin 20 were detected in infected and mock-infected colonic samples, confirming that the ratios of epithelial cells in the colonic extracts from these groups were consistent and did not account for the reduced levels of PPARγ (Fig. S3). These results were consistent with previous reports indicating a protective role of PPARγ in the intestinal epithelium in experimental inflammatory bowel disease [20]. However, the molecular mechanisms underlying these observations remain elusive.

### Depletion of epithelial PPARγ in the colon triggers an elevated immune response during infectious colitis

**Figure 3:**
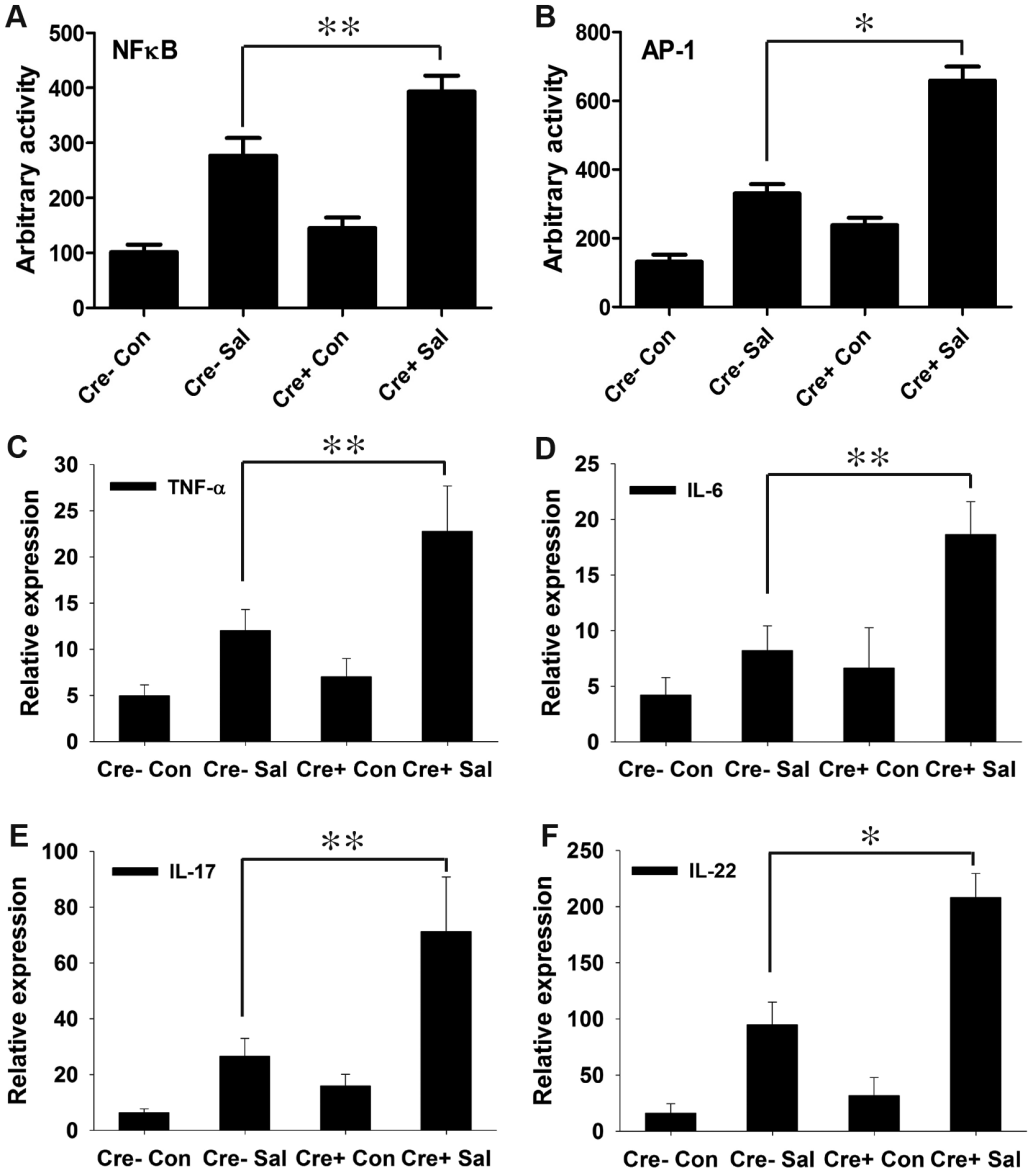
PPARγ depletion from intestinal epithelium augments the immune response to *S.* Typhimurium infection. (A and B) Nuclear extracts from colonic scrapings of mock (Con)- or *S.* Typhimurium (Sal)-infected PPARγVillinCre+ (Cre+) or littermate control PPARγVillinCre− (Cre−) mice were assessed for NFκB (A) and AP-1 (B) activities by electromobility shift assay (6–8 mice per group). The expression levels of TNF-α (C), IL-6 (D), IL-17 (E), and IL-22 (F) in the colons of mock- or *S.* Typhimurium-infected PPARγVillinCre+ or PPARγVillinCre− mice were measured by real-time PCR. Error bars = ± standard error of the mean. *p<0.001, **p<0.01.

We previously demonstrated the direct participation of PPARγ in host-microbe crosstalk and the consequent regulation of innate immune functions [35], [36]. Moreover, PPARγ has been proposed to regulate inflammation by antagonizing the NFκB and AP-1 pathways [23], [24], which in turn may modulate immune responses. To test this hypothesis in infectious colitis, we analyzed the activities of NFκB and AP-1 via electromobility shift assay using nuclear extracts from colonic scrapings. As anticipated, depletion of epithelial PPARγ was coupled with a marked increase in the activities of NFκB and AP-1 in the colon after *S.* Typhimurium infection compared to littermate control (PPARγVillinCre-) mice (Fig. 3A and B, Fig. S4).

A potential challenge was to obtain maximal numbers of epithelial cells in colonic scrapings; epithelial cells contribute the majority of colonic PPARγ, but macrophages and B and T lymphocytes also produce it [20], [22]. To overcome this problem, we used mucosal scrapings from the colon and tested for the presence of PPARγ transcripts through real-time PCR. Minimal expression and activity of PPARγ was detected in mock-infected and infected PPARγVillinCre+ mice compared to littermate control PPARγVillinCre- and wild-type (C57BL/6) mice (Fig. S5A and B). This finding was further validated in cecum scrapings (Fig. S1D). Thus, it was evident that epithelial cells were the major cell type in our samples; the presence of infiltrating macrophages and T and B cells in the colonic scrapings was possible, but minimally contributed to PPARγ production. Furthermore, no significant differences in the expression or activity of PPARγ were detected between PPARγVillinCre- and wild-type C57BL/6 mice (Fig. S5), justifying the use of PPARγVillinCre- mice as controls in subsequent experiments.

**Figure 4:**
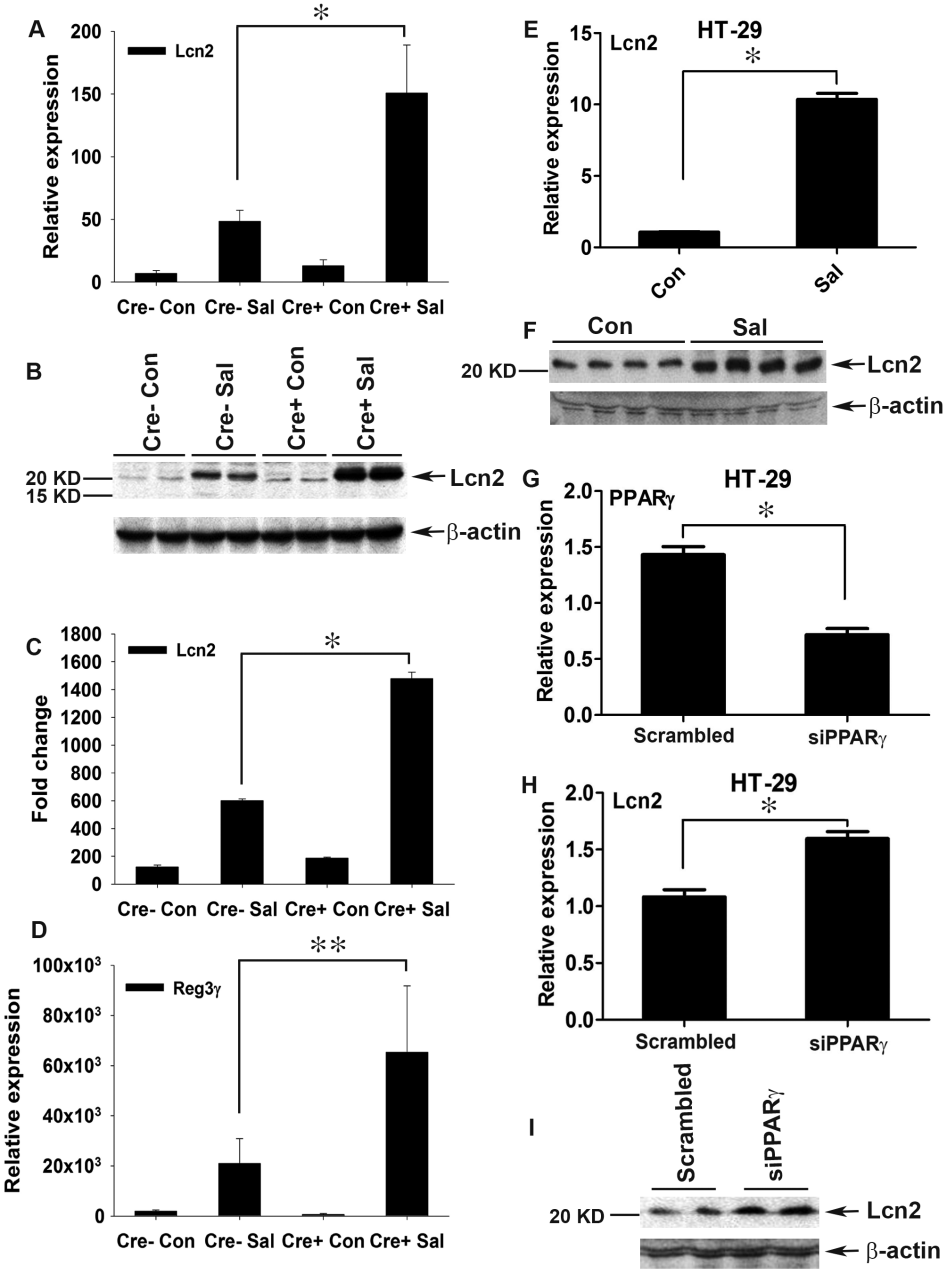
Colonic Lcn2 expression in PPARγVillinCre+ mice increases after *S.* Typhimurium challenge. (A and B) Expression levels of Lcn2 in the colons of mock (Con)- or *S.* Typhimurium (SaI)-infected PPARγVillinCre+ (Cre+) or PPARγVillinCre− (Cre−) mice (6–8 mice per group) were measured by real-time PCR (A) and by immunoblotting (B). (C) Quantitation of changes in protein expression from the immunoblot in panel B and from another representative blot from independent experiments. (D) Expression levels of Reg3γ in the colons of mock- or *S.* Typhimurium-infected mice were measured by real-time PCR. (E and F) HT-29 cells were mock- or *S.* Typhimurium-infected for 6 h, incubated for another 18 h without the pathogen, and Lcn2 expression (E) and secretion (F) were analyzed by real-time PCR and immunoblotting, respectively. (G–I) HT-29 cells were treated with siRNA directed against PPARγ, and the expression of PPARγ (G) and Lcn2 (H) in uninfected cells was analyzed by real-time PCR. The secretion of Lcn2 was analyzed by immunoblotting using concentrated culture supernatant (I). Error bars = ± standard error of the mean. *p<0.005, **p<0.01.

We next investigated the effect of increased NFκB and AP-1 activities on key regulators of inflammation. After infection, the expression levels of TNF-α and IL-6 in the colon were two-fold higher in PPARγVillinCre+ mice compared to PPARγVillinCre- mice (Fig. 3C and D). IL-6 is a key regulator of the innate T helper type 17 (T_H_17) response, a critical component of mucosal immunity to intestinal pathogens [3]. Consistent with this role, we observed substantial increases in the expression of IL-17 and IL-22, a typical innate T_H_17 response signature, in infected PPARγVillinCre+ mice compared to infected PPARγVillinCre- mice, as assessed via real-time PCR (Fig. 3E and F). IL-17 and IL-22 have been linked with intestinal innate epithelial defense mechanisms through the production of antimicrobial peptides [3], [5]–[7]. The expression of Lcn2, a principal target of IL-17 and IL-22 [3], [4], increased by approximately 7-fold in the colons of *S.* Typhimurium-infected PPARγVillinCre- mice compared to mock-infected mice, an increase that rose to a striking ∼21-fold change in infected PPARγVillinCre+ mice (Fig. 4A). This increase in Lcn2 expression in infected colons was also noticeable at the protein level (Fig. 4B and C), further confirming a hyper-magnified Lcn2 response in infected PPARγVillinCre+ mice. Lcn2 was similarly elevated in the cecum of these mice (Fig. S1E). Interestingly, TLR-4^−/−^ mice infected with *S.* Typhimurium also showed a significant increase in Lcn2 expression in the colon compared to mock-infected mice (Fig. S2H).

The expression of regenerating islet-derived 3 gamma (Reg3γ), another potent member of the host antimicrobial arsenal and a potential target of IL-17 and IL-22 [3], [37], was also tested. The expression of Reg3γ in colon followed a pattern similar to that of Lcn2 in PPARγVillinCre- and PPARγVillinCre+ mice (Fig. 4D), thus establishing a heightened innate immune response to *S.* Typhimurium in the absence of epithelial PPARγ. Moreover, *S.* Typhimurium induced a significant increase in the expression and secretion of Lcn2 (Fig. 4E and F) in parallel with PPARγ down-regulation (Fig. 1G) in colonic epithelial cells, further confirming the importance of epithelial cells in this process. To test whether this Lcn2 up-regulation was a cell-autonomous effect of PPARγ in epithelial cells, we next applied PPARγ small interfering RNA (siRNA) to HT-29 cells. A ∼50% reduction in PPARγ levels (Fig. 4G) led to a significant increase in Lcn2 expression and secretion in the absence of *S.* Typhimurium infection (Fig. 4H and I). Moreover, when these PPARγ-siRNA treated HT-29 cells were infected with *S.* Typhimurium, the expression profile of Lcn2 resembled to that observed *in vivo* (Fig. S2I). These observations not only confirmed the existence of a direct link between PPARγ and Lcn2, but also suggested that Lcn2 regulation by PPARγ may occur locally in epithelial cells without the intervention of other cell types.

At this juncture, the observed dichotomy in PPARγ's role in infectious colitis was surprising. Although we initially observed that the absence of PPARγ led to increased colitis severity (Fig. 1H and I; Fig. 2), in sharp contrast we also detected a simultaneous elevated innate immune response that seemed to serve a protective role. Taken together, these results reflect PPARγ's tight control over intestinal homeostasis in the host during *S.* Typhimurium pathogenesis. We next questioned the rationale behind this heightened Lcn2 expression, given that *S.* Typhimurium is typically resistant to Lcn2's antimicrobial activity [4], [13], [15], [16], and speculated that Lcn2 may have a more diverse role in the disease process.

### Lcn2 promotes MMP-9 stability and contributes to colitis severity

**Figure 5:**
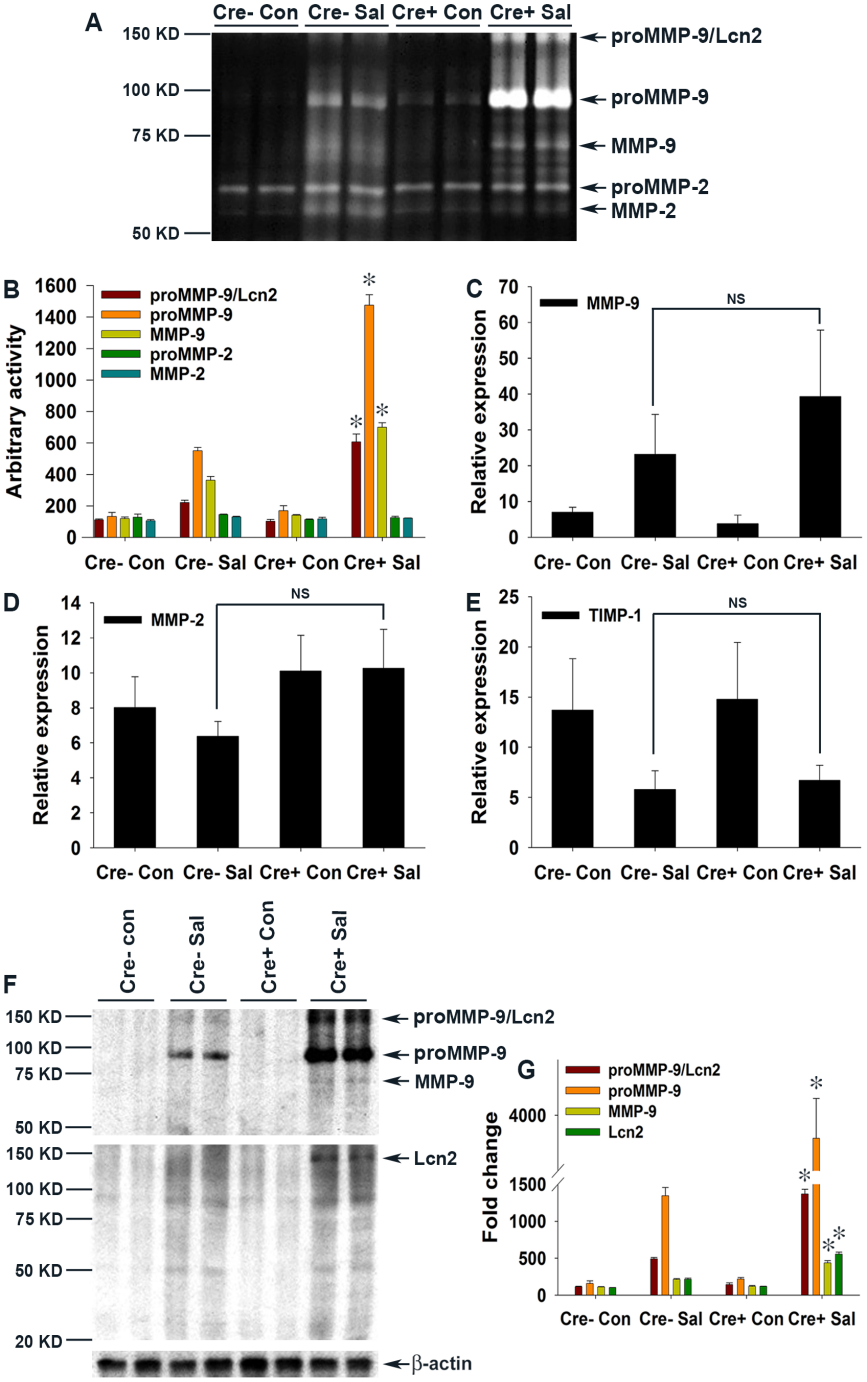
Elevated levels of Lcn2 in the colonic milieu of *S.* Typhimurium-infected mice stabilize proMMP-9. (A) The activities of MMP-9 and MMP-2 in gelatin-agarose-purified PBS (secreted) extracts of mock (Con)- or *S.* Typhimurium (SaI)-infected PPARγVillinCre+ (Cre+) or PPARγVillinCre− (Cre−) mouse colonic tissues were analyzed by gelatin zymography (6–8 mice per group). (B) Quantitation of gelatinolytic activities from the zymogram in panel A and from two other representative zymograms from independent experiments. Expression levels of MMP-9 (C), MMP-2 (D), and TIMP-1 (E) in the colons of the respective groups were measured by real-time PCR. (F) Protein levels of MMP-9 and Lcn2 in the colons of the respective mouse groups were assessed by immunoblotting using purified PBS extracts under non-reducing conditions. Flow-through from gelatin-agarose purification was probed for β-actin as a loading control. (G) Quantitation of changes in protein levels from the immunoblot in panel F and from another representative blot from independent experiments. Error bars = ± standard error of the mean. *p<0.001 vs. PPARγVillinCre- mice infected with *S*. Typhimurium. NS, not significant.

Having established that Lcn2 expression was markedly increased in PPARγVillinCre+ mice (Fig. 4A–C), we next sought to dissect its possible role in the increased colonic damage observed in PPARγVillinCre+ mice in the absence of PPARγ (Fig. 2), suggesting ongoing exaggerated protease action. Interestingly, Lcn2 has been shown to increase the stability of MMP-9 by protecting it from degradation, resulting in an increase in its enzymatic activity independent of transcriptional regulation [8], [10], [38], [39]. To test this hypothesis, we analyzed the activity of gelatin agarose-purified secreted gelatinases by zymography using phosphate-buffered saline (PBS) extracts of colonic scrapings of mock- or *S.* Typhimurium-infected mice. Interestingly, we detected a ∼140 KDa band representative of Lcn2-bound proMMP-9 (proMMP-9/Lcn2) in *S.* Typhimurium-infected PPARγVillinCre- mice that peaked to an ∼6-fold increase in infected PPARγVillinCre+ mice (Fig. 5A and B). Surprisingly, this increase in the proMMP-9/Lcn2 band in PPARγVillinCre+ mice was associated with a massive increase in the activity of proMMP-9 (detected by zymography Fig. 5A), indicating an increased stability of proMMP-9. The activity of proMMP-9 increased by ∼6 fold in PPARγVillinCre- mice and ∼15 fold in PPARγVillinCre+ mice after infection, while MMP-9 activity rose from ∼4 fold in PPARγVillinCre- mice to ∼7 fold in PPARγVillinCre+ mice after infection (Fig. 5A and B). In contrast, MMP-2 activity remained almost unchanged between groups. Importantly, no significant difference in MMP-9 gene expression occurred between the PPARγVillinCre- and PPARγVillinCre+ infected groups, as detected by real-time PCR (Fig. 5C), which confirmed that the differences in MMP-9 activity were independent of transcriptional regulation. Furthermore, MMP-2 expression was similar between the infected and mock-infected groups (Fig. 5D). No noticeable difference in the expression of TIMP-1, the endogenous inhibitor of MMP-9, was observed between the infected mice groups (Fig. 5E), eliminating the possibility of its involvement in the observed deregulation of MMP-9 activity.

To further confirm this phenomenon, we next analyzed the protein levels of MMP-9 and Lcn2 by immunoblotting gelatin-agarose-purified PBS extracts of colonic scrapings under non-reducing conditions. As anticipated, the ∼140 KDa proMMP-9/Lcn2 band, the level of which increased by ∼5 fold in infected PPARγVillinCre- mice, peaked at ∼15-fold in PPARγVillinCre+ infected mice (Fig. 5F and G). This increase led to an ∼15-fold increase in proMMP-9 protein levels in PPARγVillinCre- infected mice and reached a striking increase of ∼40 fold in PPARγVillinCre+ infected mice, while MMP-9 climbed ∼5 fold in PPARγVillinCre+ infected mice (Fig. 5F and G). When the gels were re-probed for Lcn2, bands at exactly the same location as the ∼140 KDa band, characteristic of proMMP-9 bound to Lcn2, were detected (Fig. 5F and G). These results indicate that Lcn2 is secreted from the intestinal epithelium, in accordance with previous reports [3], and more importantly confirm the binding of Lcn2 to secreted proMMP-9 in the intestinal milieu, which is pivotal for subsequent pathological processes. However, since the increase in the levels of the Lcn2/MMP-9 complex in the intestinal milieu correlates with the increased infiltration of neutrophils it is possible that these cells may contribute to the observed elevation of Lcn2/MMP-9. It is noteworthy that the ∼6-fold increase in Lcn2 secretion in *S.* Typhimurium-infected PPARγVillinCre+ mice (Fig. 5F and G) did not precisely correspond to its ∼15-fold increase at the expression level (Fig. 4A–C), because it represented only the Lcn2 fraction bound to proMMP-9 during gelatin-agarose purification of secreted gelatinases. Reasonably, no low molecular-weight band for Lcn2 alone was detected (Fig. 5F). It may also be noted that the proportion of bound or free Lcn2 or proMMP-9 at any given point would largely depend on the availability, stability, and importantly the stoichiometry of binding between these molecules. Taken together, these results conclusively indicate that lack of epithelial PPARγ substantially elevates Lcn2 expression and its secretion in the intestinal milieu during *S.* Typhimurium infection, resulting in increased MMP-9 stabilization and activity.

### Absence of Lcn2 significantly protects mice from *S.* Typhimurium-induced colitis

**Figure 6:**
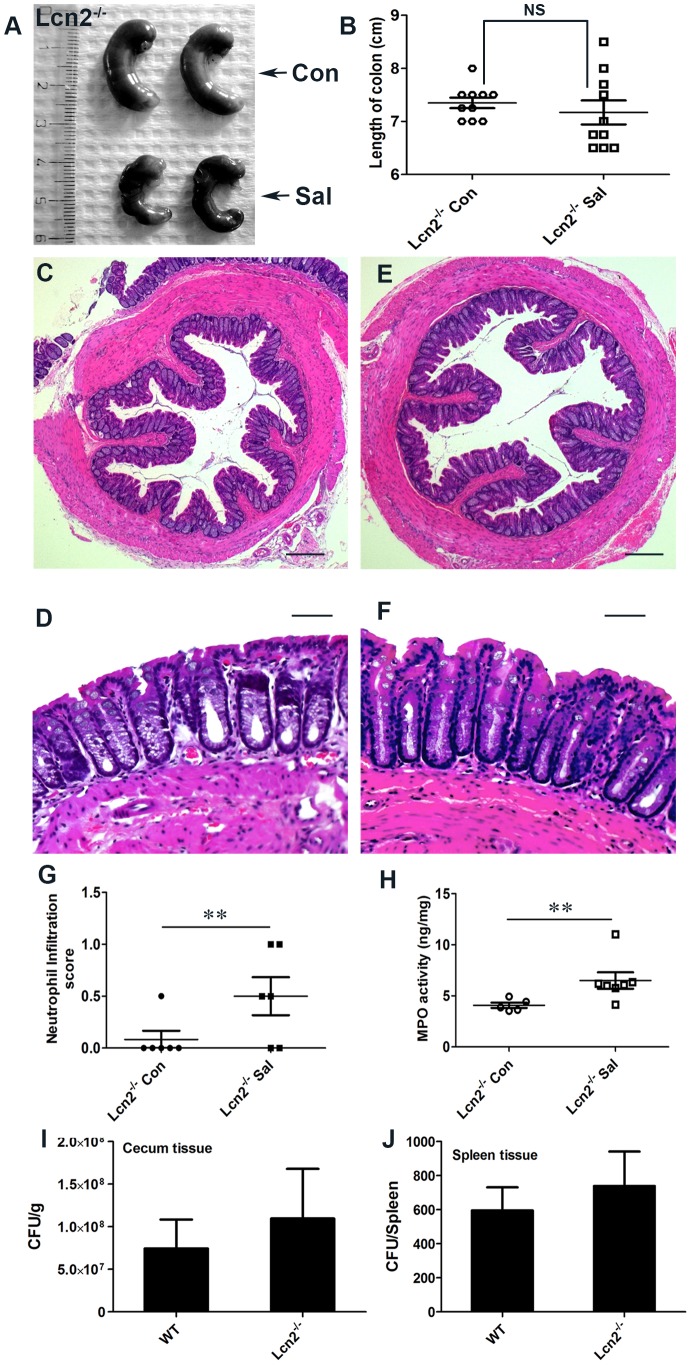
Lcn2^−/−^ mice are markedly protected from *S.* Typhimurium-induced colitis. (A and B) Groups of 8–10-week-old, streptomycin-pretreated Lcn2^−/−^ mice were mock (Con)- or *S.* Typhimurium (Sal)-infected and sacrificed after 24 h (10 mice per group). (A) Macroscopic image of whole cecum 24 h after infection. (B) Quantitation of colon lengths in mock- or *S.* Typhimurium-infected Lcn2^−/−^ mice. (C–F) Sections of colon from mock- or *S.* Typhimurium-infected Lcn2^−/−^ mice were stained with hematoxylin and eosin (scale bars, 100 µm). Lcn2^−/−^ mice mock-infected (C and D), or infected with *S.* Typhimurium (E and F). Pathology scoring was performed for neutrophil infiltration (G). (H) Myeloperoxidase (MPO) activity was determined in the colonic extracts of mice, as measured per mg of total protein. Recovery of *S.* Typhimurium from cecum tissue (I) and spleen (J), 24 h after infection. Error bars = ± standard error of the mean. **p<0.05. NS, not significant.

To validate the observed involvement of Lcn2 in *S.* Typhimurium-induced colitis, we next checked for colitis induction in streptomycin-pretreated Lcn2^−/−^ mice mock- or *S.* Typhimurium-infected for 24 h. As expected from the above observations, marked reductions in the extent and severity of *S.* Typhimurium-induced colitis were observed in mice devoid of Lcn2 (Fig. 6A and B). Shortening and thickening of the cecum and colon were considerably restricted in the Lcn2^−/−^ mice 24 h after *S.* Typhimurium challenge (Fig. 6A and B). These results were confirmed by observations of reduced thickening of the mucosa and sub-mucosa, with negligible tissue damage (Fig. 6C–H). However, a modest increase in infiltrating cells and consequent myeloperoxidase (MPO) activity was noted (Fig. 6G and H). Moreover, the number of *S.* Typhimurium in the cecum and spleen of these Lcn2^−/−^ mice was comparable to that of wild-type mice after 24 h (Fig. 6I and J). As expected, Lcn2 expression was not detected in these mice by real-time PCR (Fig. S6A) or by immunoblotting (Fig. S6B) of the colons of Lcn2^−/−^ mice. Thus, these observations highlight Lcn2's unique role in the induction and severity of infectious colitis.

**Figure 7:**
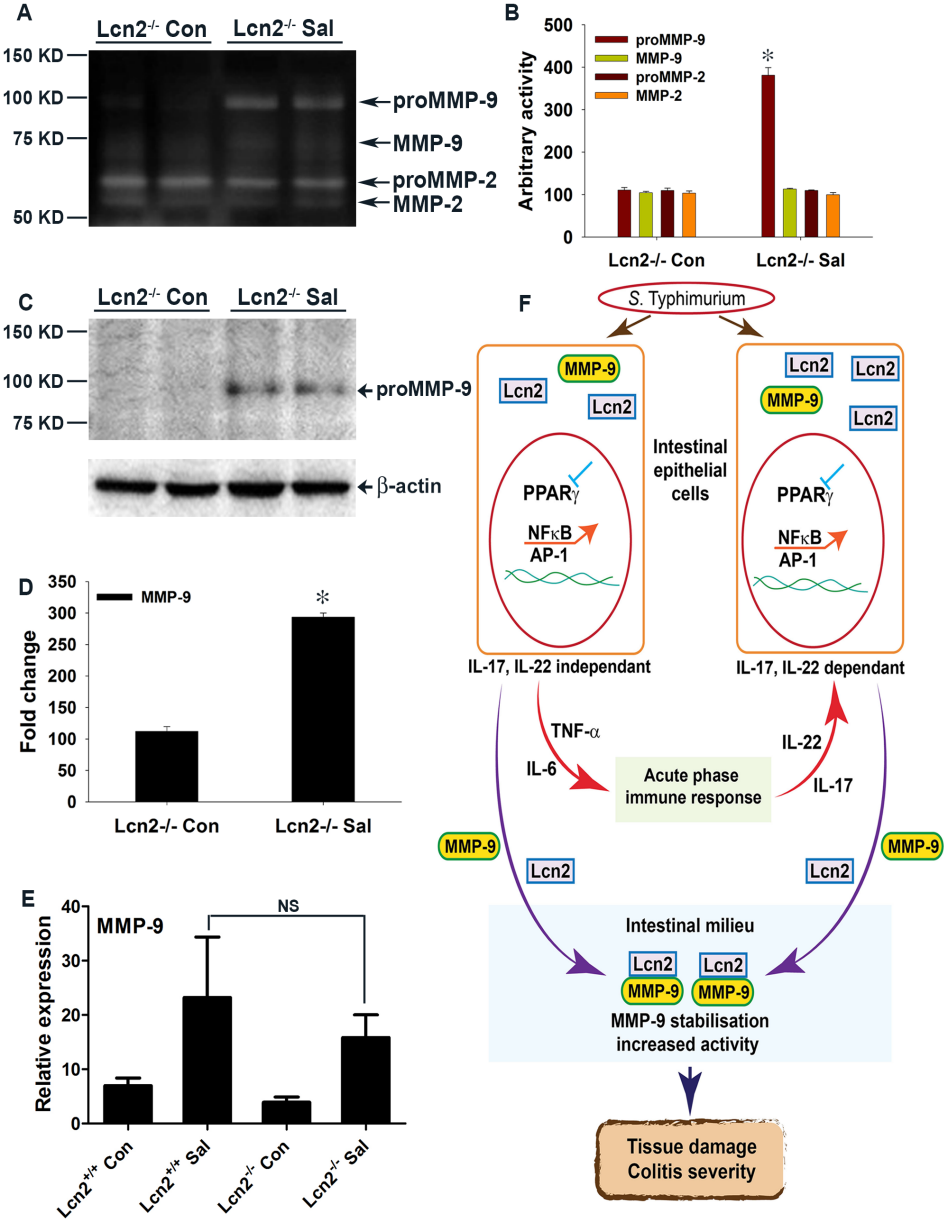
Mechanism of *S.* Typhimurium-induced intestinal damage during colitis. (A) Secretion of MMP-9 and MMP-2 in the colons of mock (Con)- or *S.* Typhimurium (SaI)-infected Lcn2^−/−^ mice was analyzed by gelatin zymography using gelatin-agarose-purified PBS extracts (6–8 mice per group). (B) Quantitation of gelatinolytic activities from the zymogram in panel A and from another representative zymogram from independent experiments. (C) MMP-9 protein levels in the colons of mice from the respective groups were assessed by immunoblotting purified PBS extracts under non-reducing conditions. Flow-through from the purification was used as a loading control. (D) Quantitation of changes in protein levels from the immunoblot in panel C and from another representative blot from independent experiments. (E) MMP-9 expression levels in the colons of mock- or *S.* Typhimurium-infected Lcn2^+/+^ and Lcn2^−/−^ mice were measured by real-time PCR. Error bars = ± standard error of the mean. *p<0.0001 vs. Lcn2^−/−^ control. NS, not significant. (F) *S.* Typhimurium down-regulates PPARγ in intestinal epithelial cells. The subsequent decrease in PPARγ activity leads to the activation of NFκB and AP-1 and the release of TNF-α and IL-6. Through activation of the acute-phase immune response, TNF-α and IL-6 induce IL-17 and IL-22. NFκB and AP-1 activation either directly or in conjunction with IL-17 and IL-22 induce Lcn2 expression in epithelial cells and its consequent secretion into the intestinal milieu. Extracellular binding of Lcn2 to secreted MMP-9 increases MMP-9 stabilization and activity, resulting in extensive tissue damage during infectious colitis.

To more precisely track the molecular mechanisms active in Lcn2^−/−^ mice after *S.* Typhimurium infection, we assessed the secretion of gelatinases in the colon. We observed an ∼3.5-fold increase in proMMP-9 activity in Lcn2^−/−^ mice after infection versus mock infection (Fig. 7A and B), compared to a ∼6-fold increase in PPARγVillinCre- infected mice (Fig. 5A and B). No notable differences in the activities of MMP-9 and MMP-2 were detected between groups. The basal secretion of gelatinases was similar in PPARγVillinCre- and Lcn2^−/−^ mock-infected mice (Fig. S7A), justifying the comparison between these groups in this case.

We next examined the levels of secreted MMP-9 using gelatin-purified colonic extracts of mock- or *S.* Typhimurium-infected Lcn2^−/−^ mice. Compared to the ∼15-fold increase in secreted proMMP-9 that was observed in PPARγVillinCre- mice after infection (Fig. 5F and G), a mere ∼3-fold increase in the levels of secreted proMMP-9 was detected in Lcn2^−/−^ mice after infection, suggesting reduced extracellular stability and possible degradation of MMP-9 protein in the absence of Lcn2 (Fig. 7C and D). No significant difference in the colonic expression of MMP-9 was detected between Lcn2^−/−^ and Lcn2^+/+^ mice after infection (Fig. 7E), confirming that the differences in the protein levels of MMP-9 were due to impaired stability. Moreover, colonic expression of MMP-2 and TIMP-1 was similar in Lcn2^+/+^ and Lcn2^−/−^ mice after infection (Fig. S7B and C), excluding their involvement in this process.

To investigate any possible mechanistic differences between the Lcn2^−/−^ and Lcn2^+/+^ mice during *S.* Typhimurium infection, we validated several key regulators involved in the pathophysiology of infectious colitis. Real-time PCR revealed no appreciable differences in the down-regulation of PPARγ by *S.* Typhimurium between Lcn2^−/−^ and Lcn2^+/+^ mice (Fig. S8A), confirming that PPARγ regulation by *S.* Typhimurium was essentially the same in Lcn2^−/−^ mice. Consequently, the expression levels of TNF-α and IL-6 were similar between the infected groups (Fig. S8B and C). Colonic expression of IL-17 and IL-22 also exhibited negligible differences between Lcn2^−/−^ and Lcn2^+/+^ infected mice (Fig. S8D and E), as did Reg3γ expression (Fig. S8F). Together, these observations suggest that the mechanistic chain of events during *S.* Typhimurium infection in wild-type and Lcn2^−/−^ mice was fundamentally similar; the absence of Lcn2 exclusively conferred protection to these mice against *S.* Typhimurium-induced colitis.

To determine whether the regulation of PPARγby *S.* Typhimurium is a transient effect or whether the Lcn2^−/−^ mice are protected at later stages of infection, we infected PPARγVillinCre-, PPARγVillinCre+, Lcn2^−/−^, and Lcn2^+/+^ mice with *S.* Typhimurium and sacrificed them after 72 h. Long-term *S.* Typhimurium infection resulted in more severe colitis in PPARγVillinCre+ mice compared to PPARγVillinCre- mice, while Lcn2^−/−^ mice were significantly protected against colitis (Fig. S9A); colonic shortening displayed a similar profile (Fig. S9B). There was no significant difference in the number of *S.* Typhimurium recovered from the cecum or spleen of these mice 72 h after infection (Fig. S9C and D). Histological analysis revealed more severe colitis in PPARγVillinCre+ mice than in PPARγVillinCre- mice, with significantly increased neutrophil infiltration and MPO activity (Fig. S9E-L and S9U-W). Although the Lcn2^−/−^ mice exhibited moderate colitis, they were associated with marked reductions in neutrophil infiltration, edema, and MPO activity compared to the rest of the groups 72 h after infection (Fig. S9M–W). Interestingly, PPARγ expression was still significantly reduced after 72 h in the colons of PPARγVillinCre- mice, confirming that PPARγ regulation by *S.* Typhimurium was not a transient event (Fig. S9X and Y).

## Discussion

Intestinal pathogens employ diverse strategies to modulate the host environment in order to survive in this competitive niche. The approaches that individual pathogens adopt depend largely on the tenure of their residence in the host. For instance, *H. pylori* up-regulates host PPARγ as part of a feedback mechanism to suppress exaggerated inflammation, ensuring its unperturbed long-term survival in the host [17], [19]. Similarly, *M. tuberculosis* induces PPARγ expression in infected individuals and subsequently interacts with host PPARγ by modulating macrophage function for its survival [18]. Here, we unravel a novel mechanism used by *S.* Typhimurium to down-regulate PPARγ in the intestinal epithelium, initiating acute inflammation via the host immune and protease machinery, thereby transforming the intestine into a more hostile niche where it is best adapted to survive and outgrow its competitors. This TLR-4-independent regulation of PPARγ by *S.* Typhimurium seems to be characteristic of this pathogen, since *C. rodentium* infection, did not alter PPARγ levels.

PPARγ activation has been shown to ameliorate the severity of inflammatory bowel disease in rodent DSS, trinitrobenzene sulphonic acid, and ischemic colitis models [20], [27], [30], [32], [33]. Since PPARγ is expressed in epithelial cells as well as in immune cells infiltrating colonic tissue during inflammation, the cell type that mainly contributes to PPARγ production during colitis remains a point of contention. Interestingly, clinical reports indicate that PPARγ expression in colonic epithelium is impaired in ulcerative colitis patients, while its expression in inflammatory cells remains normal [40]. This observation was corroborated by Adachi et al., who reported that PPARγ expressed in the colonic epithelium has an endogenous role in protection against DSS-induced colitis [20]. These findings, together with our data in Fig. 1G, demonstrating PPARγ regulation by *S.* Typhimurium in human colonic epithelial cells, prompted us to use epithelial-specific PPARγ-null mice to unravel the chain of events that occur during *S.* Typhimurium-induced colitis. This strategy allowed us to magnify the subtle molecular changes induced by *S.* Typhimurium in the host via down-regulation of epithelial PPARγ. *S.* Typhimurium induced much more severe colitis in these mice (Fig. 1H and I; Fig. 2), highlighting, for the first time, the importance of intestinal epithelium-derived PPARγ in protection against bacterial pathogenesis.

Our data demonstrate that *S.* Typhimurium-induced depletion of epithelial PPARγ uncouples PPARγ's tight control over the inflammatory transcription factors NFκB and AP-1, resulting in the release of the pro-inflammatory cytokines TNF-α and IL-6. This influx of pro-inflammatory cytokines from the intestinal epithelium initiates an acute-phase immune response characterized by elevated expression of IL-17 and IL-22. These results are consistent with a recent report by Geddes et al. of the induction of the innate T_H_17 response by *S.* Typhimurium during the early phases of infection [3]. Collectively, our results imply that these inflammatory signaling circuits are orchestrated by *S.* Typhimurium during the early phases of infection via the regulation of epithelial PPARγ, which is pivotal for the entire process.

The secretion of IL-17 and IL-22 in the inflamed colon, which is initiated by *S.* Typhimurium, has been shown to facilitate the production of antimicrobials, including Lcn2, from the intestinal epithelium [3]–[7]. Our observations confirm the contribution of IL-17 and IL-22 to Lcn2 production and secretion, but also suggest that *S.* Typhimurium-induced increases in NFκB and AP-1 activity in epithelial cells via PPARγ down-regulation may directly influence Lcn2 expression in the same cells independent of IL-17 and IL-22 (Fig. 7F). This hypothesis was further corroborated by the induction of Lcn2 in non-infected human colonic epithelial cells treated with siRNA directed against PPARγ. It seems reasonable to assume that *S.* Typhimurium may utilize this more direct pathway of Lcn2 regulation, since Lcn2 expression is known to be regulated by NFκB or even AP-1 [41], [42]; importantly, this entire sequence of events may occur in affected epithelial cells.

Understanding the rationale behind the substantial increase in Lcn2 secretion from the intestinal epithelium during *S.* Typhimurium infection, which typically utilizes salmochalin, a siderophore resistant to Lcn2 action [4], [13], [15], [16], for iron uptake, was perhaps the biggest challenge in this study. Here, we documented that *S.* Typhimurium-induced elevated influx of secreted Lcn2 in the intestinal milieu leads to stabilization and a significant increase in MMP-9 activity, through direct extracellular protein-protein binding (Fig. 7F). MMP-9, a member of a family of zinc-dependent endopeptidases that have broad substrate specificity, has been shown to play a pivotal role in the degradation and remodeling of the extracellular matrix during bacterial pathogenesis [19], [43]. Moreover, MMP-9-null mice exposed to DSS or to *S.* Typhimurium were previously significantly protected from colitis [44], [45], confirming MMP-9's importance in the etiology of the disease. Although MMPs are secreted by a variety of cell types, such as fibroblasts, epithelial cells, endothelial cells, neutrophils, macrophages, and lymphocytes, MMP-9 is predominantly expressed in epithelial cells and in inflammatory cells during colitis [44], [46]. Our data suggest that this crosstalk between Lcn2 and MMP-9 in the inflamed gut, which results in increased MMP-9 activity, was crucial for the deleterious impact on the intestinal mucosa observed during infectious colitis. This novel mechanism by which *S.* Typhimurium exploits the host Lcn2 and MMP-9 synergy to aggravate inflammation and colitis severity is critical, as it calls for the reinterpretation of studies on microbial pathogenesis; other potential pathogens may also employ similar mechanisms.

Interestingly, mice lacking Lcn2 were considerably protected against *S.* Typhimurium-induced colitis even at the later stages of infection, confirming the key role of this secreted protein in *S.* Typhimurium pathogenesis. We detected no significant differences in the overall mechanism acting in Lcn2-null and wild-type infected mice, with the exception of a decrease in MMP-9 stability and activity in the colon. Our observations of Lcn2-null mice exposed to *S.* Typhimurium, which indicated increased expression of IL-17, IL22, and TNF-α, are consistent with the findings of Raffatellu et al. [4]. We also noted moderate inflammation in the colon during the later stages of infection. However, disease severity may be impacted by differences in experimental setup, including *S.* Typhimurium strains, bacterial load and phase of growth in the inoculum, and importantly, differences in intestinal microflora between mice.

In conclusion, our investigation unveiled a novel pathogenic mechanism utilized by *S.* Typhimurium to thrive and to induce colitis in its host. This study motivates the development of therapeutic interventions directed against this Lcn2-dependent, MMP-9-driven tissue degradation pathway to combat salmonellosis. However, research aimed toward a better understanding of the pathogenic mechanisms of *S.* Typhimurium or other pathogens in the gut remains an exciting area for future studies.

## Materials and Methods

### Ethics statement

All protocols involving animals were approved by the Regional Animal Research Ethical Board, Stockholm, Sweden, following proceedings described in European Union legislation (Council Directive 86/609/EEC). Animal husbandry was in accordance with institutional guidelines at Karolinska Institutet and was approved by the above-mentioned ethical board (Stockholms norra djurförsöksetiska nämnd, Ref: N 100/10).

### Bacterial strains, culture and colonization assessment

The naturally streptomycin-resistant wild type strain *S. enterica* serovar Typhimurium SL1344 [47], a generous gift from Prof. Mikael Rhen was used in this study. Naturally occurring naldixic acid-resistant *Citrobacter rodentium*, DBS100 (ATCC 51459) was also used for mouse infection. Prior to inoculation into host mice, strain SL1344 and *C. rodentium* were grown overnight at 37°C in Luria-Bertani (LB) broth, diluted 1∶20 in fresh medium, and sub-cultured for 3–4 h under mild aeration until an optical density of 0.4 to 0.6 at 600 nm was reached. Bacteria were washed twice in cold PBS and then suspended in cold PBS for mouse inoculation.

The cecum and spleen from post-sacrifice mice were collected in 1 mL of sterile PBS. Samples were kept on ice, minced, and homogenized. Serial dilutions of the homogenates were plated on LB agar plates supplemented with 100 µg/mL streptomycin to enumerate *S.* Typhimurium. Plates were incubated overnight at 37°C, and colonies were counted thereafter.

### Cell-culture assays

The human epithelial cell line HT-29 (ATCC-HTB-38) was obtained from the American Type Culture Collection. Cells were grown in RPMI 1640 (Invitrogen) medium supplemented with 10% heat-inactivated fetal calf serum (Invitrogen). Cells were maintained in a 37°C humidified atmosphere with 5% CO_2_. Epithelial morphogenesis was monitored via microscopy; cell densities for each experiment did not exceed 80% to prevent contact inhibition.

For co-culture experiments, cells were treated with *S.* Typhimurium (0.25×10^7^ cells/well; 10∶1 bacterial cells:eukaryotic cells) for 6 h. Controls were treated with culture medium only. After 6 h medium was removed, cells were washed, fresh medium with 1% penicillin/streptomycin was added, and the cells were incubated for 18 h, after which the cells were collected and lysed.

For siRNA experiments HT-29 cells were plated at a density of 0.0625×10^5^ cells/cm^2^. Down-regulation of PPARγ transcripts was achieved with SMART Pool siRNA directed against PPARγ (Thermo Scientific). Controls were transfected with non-targeting siRNA (Thermo Scientific) at a final siRNA concentration of 40 nM. Transfection was carried out according to the manufacturer's protocol using DharmaFECT 4 (Thermo Scientific) reagent at a final concentration of 0.3%.

### Bacterial infection in mice

Specific pathogen-free C57BL/6 wild-type mice carrying a targeted disruption of the gene encoding PPARγ in intestinal epithelial cells were generated by breeding animals harboring a floxed *Pparγ* (PPAR*γ* ^fl/fl^) [48] to mice expressing the *Cre* transgene under control of the *villin* promoter; these mice were designated as PPARγVillinCre+, and their littermate control mice were designated as PPARγVillinCre−. Lcn2-deficient (Lcn2^−/−^) mice [11], [38] and TLR4^−/−^ mice (Jackson Laboratory Stock No: 007227) aged 8–10 weeks were also used in this study. The Lcn2^−/−^ mice were generated previously [11] and were backcrossed into the C57BL/6 background for at least 10 generations [38]. All experiments were performed under standard controlled conditions and all efforts were made to minimize animal suffering.

Groups of mice were pretreated with streptomycin (0.1 mL of a 200 mg/mL solution in sterile water) orally 24 h prior to either mock (PBS) or *S.* Typhimurium (1×10^8^ colony-forming units/mouse) inoculation via gavage. At 24 h or 72 h after infection, mice were euthanized and the cecum, spleen, and colon were collected for analysis. For *C. rodentium* infection, mice were given metronidazole at 750 mg/L for 4 days, which was withdrawn prior to inoculation with 1×10^9^ colony-forming units/mouse via gavage. *C. rodentium*-infected mice were sacrificed 6 days after infection [49]. Colon lengths were measured using a centimeter scale.

### Tissue extraction, partial purification of gelatinases, and zymography

The mucosal layer of the colon was carefully scraped and suspended in PBS containing protease inhibitors (Roche), minced, and centrifuged at 6000 g for 15 min. The supernatant was collected for use as PBS extracts for the analysis of secreted proteins, while the pellet was re-extracted in lysis buffer (10 mM Tris-HCl [pH 8], 150 mM NaCl, 1% Triton X-100, and protease inhibitors) to obtain Triton X-100 extracts [41]. For partial purification of MMP-9 and MMP-2, PBS extracts of the respective samples were incubated with gelatin-agarose beads (Sigma) at 4°C for 1 h followed by centrifugation at 1500 rpm. The supernatant was collected as flow-through and used as a loading control. The pellet was washed twice with PBS through centrifugation at 1500 rpm and the gelatinases were eluted in Lammeli sample loading buffer. For assays of MMP-9 and MMP-2 activity, gelatin zymography was performed as described previously [43]. Zymographic bands were quantified using LabImage software (KAPELAN).

### Immunoblotting

Triton X-100 extracts (50 µg/lane) from colon samples were immunoblotted. Gelatin-agarose-purified PBS extracts were immunoblotted under non-reducing conditions. Cell-culture supernatants were concentrated using a vacuum centrifuge and volumetrically analyzed by immunoblotting.

Immunoblots were probed with anti-PPARγ (Cell Signaling), anti-MMP-9 (Abcam), anti-Lcn2 (Abcam), and anti-β-actin (Santa Cruz Biotechnology) antibodies. Immunodetection with an appropriate secondary peroxidase-conjugated antibody (DAKO) was followed by electrochemiluminescence (Santa Cruz Biotechnology). Quantification of protein bands was performed with the LabImage software. Fold changes were calculated using densitometry values for bands representing proteins of interest, normalized to densitometry values for β-actin bands of respective samples. Representative blots from at least two independent experiments are shown.

### Real-time PCR

Total RNA was extracted with an RNeasy Mini Kit (Qiagen), and cDNA was synthesized with SuperScript II (Invitrogen), both procedures according to the manufacturers' protocols. We measured gene expressions with SYBR Green (Applied Biosystems)-based quantitative reverse transcription PCR. Primers were designed and tested according to Applied Biosystems recommendations (Table S1). Sample setups always included at least five biological replicates and experimental triplicates. The changes in mRNA expression of respective samples compared to control were expressed as ΔΔCt = ΔCt_control_−ΔCt_respective samples_ (ΔCt = Ct value for the gene of interest - Ct value for β-actin of the respective sample). Relative expressions in the genes in respective samples were calculated as 2^ΔΔCt^ [19].

### Electromobility shift assay

Nuclear extracts from colonic scrapings were prepared according the nuclear extraction protocol of Schreiber [50]. DNA binding was assayed with 10 µg of nuclear extract in binding buffer (25 mM HEPES [pH 7.9], 70 mM KCl, 10% glycerol, 5 mM dithiothreitol, and 1 µg polydIdC (Amersham)) in the presence of 50,000 cpm of a radiolabeled oligonucleotide probe. The probe for PPARγ (sequences 5′-TCTCTCTGGGTGAAATGTGC-3′ and 5′-AGAGGCACATTTCACCCAGAGAGA-3′) has high PPARγ-specificity and moderate affinity to ensure weak binding to other PPARs [35]. Probes for NFκB (sequences 5′-GATCCAGAGGGGACTTTCCGAG-3′ and 5′- TCGACTCGGAAAGTCCCCTCTG-3′) and for AP-1 (sequences 5′-CTGATGACTCAGAG-3′ and 5′-CTCTGAGTCATCAG-3′) were used. polydIdC and probe were added to extracts and incubated for 30 min before gel electrophoresis. Bands were quantified with LabImage.

### Histology and pathology scoring

The colons of mock- or *S.* Typhimurium-infected mice from the respective groups were sectioned for histological studies. Distal part of the colon was used for histological analysis in all cases. Tissue samples were fixed in 10% formalin and embedded in paraffin. Sections (5 µm) were cut with a microtome, stained with hematoxylin and eosin [43], and observed under a Zeiss microscope. Images were captured using Axiovision, LE 64 software (Carl Zeiss Microscopy) at original magnification 5×10 and 20×10 and processed in Adobe Photoshop CS6 (Adobe Systems incorporated). Pathology scoring for neutrophil infiltration and edema was rated from 0 to 5 according to severity, under blinded conditions, by an experienced pathologist (RMB).

### MPO activity assay

Whole-cell extracts from colonic scrapings were assayed for MPO activity using the Myeloperoxidase Activity Assay Kit (Invitrogen) following the manufacturer's protocol.

### Statistical analysis

Densitometry data were fitted using SigmaPlot 2001 (SPSS) or GraphPad Prism 5 (GraphPad Software). Data are presented as the mean ± standard error of the mean. Between-group comparisons were carried out using either Student's *t*-test or Student-Newman-Keuls test (ANOVA).

## Supporting Information

Figure S1 ***S.*** **Typhimurium down-regulates PPARγ in the cecum during colitis.** (A, B, and C) Groups of 8–10-week-old, streptomycin-pretreated C57BL/6 mice (WT) were mock (Con)- or *S.* Typhimurium (SaI)-infected and sacrificed after 24 h (10 mice per group). PPARγ expression in the cecum was analyzed by real-time PCR (A) and by immunoblotting (B). (C) Electromobility shift assay of PPARγ activity in nuclear extracts from the cecum. (D and E) Groups of age-matched, streptomycin-pretreated PPARγVillinCre+ (Cre+) or littermate control PPARγVillinCre− (Cre−) mice were mock- or *S.* Typhimurium-infected and sacrificed after 24 h (6–8 mice per group). PPARγ (D) and Lcn2 (E) expression in the cecum was analyzed by real-time PCR. Error bars = ± standard error of the mean. *p<0.005, **p<0.05.
(TIF)

Figure S2 ***S.*** **Typhimurium down-regulates PPARγ independent of TLR-4 signaling.** (A, B, and C) Groups of 8–10-week-old, streptomycin-pretreated C57BL/6 mice were mock (Con)- or *S.* Typhimurium (SaI)-infected and sacrificed after 24 h (10 mice per group). The expression of TLR-4 (A), TLR-2 (B), and TLR-5 (C) in the colon was analyzed by real-time PCR. (D–F and H) Age-matched, streptomycin-pretreated TLR4^−/−^ mice were mock- or *S.* Typhimurium-infected and sacrificed after 24 h (5 mice per group). Expression of TLR-4 (D), TLR-2 (E), and Lcn2 (H) in the colon was analyzed by real-time PCR. (F) Quantitation of colon lengths in the respective mouse groups. (G) Metronidazole-pretreated C57BL/6 mice were mock- or *C. rodentium*-infected, sacrificed 6 days after infection and colon lengths in the respective mice were quantified. (I) HT-29 cells were treated with siRNA directed against PPARγ, infected with *S.* Typhimurium and the expression of Lcn2 in infected cells was analyzed by real-time PCR. Error bars = ± standard error of the mean. *p<0.005.
(TIF)

Figure S3 **Analysis of epithelial cell markers in colonic scrapings.** Groups of age-matched, streptomycin-pretreated PPARγVillinCre+ (Cre+) or littermate control PPARγVillinCre− (Cre−) mice were mock (Con)- or *S.* Typhimurium (SaI)-infected and sacrificed after 24 h (6–8 mice per group). The expression levels of villin 1 (A), cytokeratin 8 (B), and cytokeratin 20 (C) in colonic scrapings were analyzed by real-time PCR. Error bars = ± standard error of the mean.
(TIF)

Figure S4 **NFκB and AP1 activity in the colons of PPARγVillinCre+ mice after** ***S.*** **Typhimurium infection.** Electromobility shift assay of NFκB activity (A) and AP-1 activity (B) in nuclear extracts from colonic scrapings of PPARγVillinCre+ (Cre+) or PPARγVillinCre− (Cre−) mice 24 h after mock (Con)- or *S.* Typhimurium (SaI)-infection (6 mice per group).
(TIF)

Figure S5 **Determination of the efficiency of tissue-specific PPARγ ablation.** Groups of 8–10-week-old, streptomycin-pretreated C57BL/6 (WT), PPARγVillinCre+ (Cre+), or littermate control PPARγVillinCre− (Cre−) mice were mock (Con)- or *S.* Typhimurium (SaI)-infected and sacrificed after 24 h (6–8 mice per group). (A) PPARγ expression in colonic scrapings was analyzed by real-time PCR. Error bars = ± standard error of the mean. *p<0.001 vs. WT or Cre- mice. NS, not significant. (B) Electromobility shift assay of PPARγ activity in nuclear extracts of colonic scrapings.
(TIF)

Figure S6 **Determination of the efficiency of the Lcn2^−/−^ mouse model.** (A and B) Groups of 8–10-week old, streptomycin-pretreated Lcn2^+/+^ and Lcn2^−/−^ mice were mock (Con)- or *S.* Typhimurium (SaI)-infected and sacrificed after 24 h (6–8 mice per group). Lcn2 expression in the colon was analyzed by real-time PCR (A) or by immunoblotting (B). Error bars = ± standard error of the mean. *p<0.005 vs. Lcn2^+/+^ control.
(TIF)

Figure S7 **Determination of basal secretion of gelatinases and expression of MMP-2 and TIMP-1 in Lcn2^−/−^ mice.** (A) Secretion of MMP-9 and MMP-2 in the colons of mock (Con)-infected PPARγVillinCre− or Lcn2^−/−^ mice (6–8 mice per group) was analyzed by gelatin zymography using gelatin-agarose-purified PBS extracts. Expression levels of MMP-2 (B) and TIMP-1 (C) in the colons of mock- or *S.* Typhimurium (SaI)-infected Lcn2^+/+^ and Lcn2^−/−^ mice were measured by real-time PCR (6–8 mice per group). Error bars = ± standard error of the mean. NS, not significant.
(TIF)

Figure S8 **Assessment of mechanistic differences between Lcn2^+/+^ and Lcn2^−/−^ mice during** ***S.*** **Typhimurium infection.** Expression levels of PPARγ (A), TNF-α (B), IL-6 (C), IL-17 (D), IL-22 (E), and Reg3γ (F) in the colons of mock (Con)- or *S.* Typhimurium (SaI)-infected Lcn2^+/+^ and Lcn2^−/−^ mice were measured by real-time PCR (6–8 mice per group). Error bars = ± standard error of the mean. NS, not significant.
(TIF)

Figure S9 **Severity of colitis 72 h after** ***S.*** **Typhimurium infection in mice.** Groups of age-matched, streptomycin-pretreated PPARγVillinCre+ (Cre+), littermate control PPARγVillinCre− (Cre−), Lcn2^−/−^, and littermate control Lcn2^+/+^ mice were mock (Con)- or *S.* Typhimurium (SaI)-infected and sacrificed after 72 h (6 mice per group). (A) Macroscopic image of whole cecum after mock or *S.* Typhimurium infection. (B) Quantitation of colon lengths. Recovery of *S.* Typhimurium from cecum tissue (C) and spleen (D) 72 h after infection. Sections of colon from these mice were stained with hematoxylin and eosin (E–T). PPARγVillinCre− mice after mock infection (E and F), or after *S.* Typhimurium infection (G and H). PPARγVillinCre+ mice after mock infection (I and J), or after infection with *S.* Typhimurium (K and L). Lcn2^+/+^ mice after mock infection (M and N), or after infection with *S.* Typhimurium (O and P). Lcn2^−/−^ mice after mock infection (Q and R), or after *S.* Typhimurium infection (S and T). All scale bars are 500 µm. Pathology scoring was carried out for neutrophil infiltration (U) and edema (V). (W) Myeloperoxidase (MPO) activity in colonic extracts from mice, measured per mg of total protein. PPARγ expression in colonic scrapings from PPARγVillinCre− mice analyzed by real-time PCR (X), and immunoblotting (Y). Error bars = ± standard error of the mean. *p<0.005, **p<0.05 vs. appropriate control or as indicated.
(TIF)

Table S1 **Details of primers used for real-time RT-PCR analysis of mouse colonic tissues and human cultured cells.** (A) The details of the mRNA of interest, sequence of primer pairs with amplicon size used for RT-PCR analysis of mouse colonic tissues. (B) The details of the mRNA of interest, sequence of primer pairs with amplicon size used for RT-PCR analysis of human cultured cells.
(DOCX)

## References

[1] Baumler AJ, Tsolis RM, Ficht TA, Adams LG (1998) Evolution of host adaptation in Salmonella enterica. Infection and Immunity 66: 4579–4587. 9746553 10.1128/iai.66.10.4579-4587.1998 PMC108564

[2] Barthel M, Hapfelmeier S, Quintanilla-Martinez L, Kremer M, Rohde M, et al (2003) Pretreatment of Mice with Streptomycin Provides a Salmonella enterica Serovar Typhimurium Colitis Model That Allows Analysis of Both Pathogen and Host. Infection and Immunity 71: 2839–2858. 12704158 10.1128/IAI.71.5.2839-2858.2003 PMC153285

[3] Geddes K, Rubino SJ, Magalhaes JG, Streutker C, Le Bourhis L, et al (2011) Identification of an innate T helper type 17 response to intestinal bacterial pathogens. Nature medicine 17: 837–844. 10.1038/nm.2391 21666695

[4] Raffatellu M, George MD, Akiyama Y, Hornsby MJ, Nuccio SP, et al (2009) Lipocalin-2 resistance confers an advantage to Salmonella enterica serotype Typhimurium for growth and survival in the inflamed intestine. Cell host & microbe 5: 476–486. 19454351 10.1016/j.chom.2009.03.011 PMC2768556

[5] Bettelli E, Korn T, Oukka M, Kuchroo VK (2008) Induction and effector functions of T(H)17 cells. Nature 453: 1051–1057. 18563156 10.1038/nature07036 PMC6280661

[6] Colonna M (2009) Interleukin-22-producing natural killer cells and lymphoid tissue inducer-like cells in mucosal immunity. Immunity 31: 15–23. 19604490 10.1016/j.immuni.2009.06.008

[7] Ouyang W, Kolls JK, Zheng Y (2008) The biological functions of T helper 17 cell effector cytokines in inflammation. Immunity 28: 454–467. 18400188 10.1016/j.immuni.2008.03.004 PMC3424508

[8] Kjeldsen L, Johnsen AH, Sengelov H, Borregaard N (1993) Isolation and primary structure of NGAL, a novel protein associated with human neutrophil gelatinase. The Journal of biological chemistry 268: 10425–10432. 7683678

[9] Triebel S, Blaser J, Reinke H, Tschesche H (1992) A 25 kDa alpha 2-microglobulin-related protein is a component of the 125 kDa form of human gelatinase. FEBS letters 314: 386–388. 1281792 10.1016/0014-5793(92)81511-j

[10] Yan L, Borregaard N, Kjeldsen L, Moses MA (2001) The high molecular weight urinary matrix metalloproteinase (MMP) activity is a complex of gelatinase B/MMP-9 and neutrophil gelatinase-associated lipocalin (NGAL). Modulation of MMP-9 activity by NGAL. The Journal of biological chemistry 276: 37258–37265. 11486009 10.1074/jbc.M106089200

[11] Berger T, Togawa A, Duncan GS, Elia AJ, You-Ten A, et al (2006) Lipocalin 2-deficient mice exhibit increased sensitivity to Escherichia coli infection but not to ischemia-reperfusion injury. Proceedings of the National Academy of Sciences of the United States of America 103: 1834–1839. 16446425 10.1073/pnas.0510847103 PMC1413671

[12] Flo TH, Smith KD, Sato S, Rodriguez DJ, Holmes MA, et al (2004) Lipocalin 2 mediates an innate immune response to bacterial infection by sequestrating iron. Nature 432: 917–921. 15531878 10.1038/nature03104

[13] Raffatellu M, Baumler AJ (2010) Salmonella's iron armor for battling the host and its microbiota. Gut microbes 1: 70–72. 21327120 10.4161/gmic.1.1.10951 PMC3035136

[14] Stecher B, Robbiani R, Walker AW, Westendorf AM, Barthel M, et al (2007) Salmonella enterica serovar typhimurium exploits inflammation to compete with the intestinal microbiota. PLoS biology 5: 2177–2189. 17760501 10.1371/journal.pbio.0050244 PMC1951780

[15] Fischbach MA, Lin H, Zhou L, Yu Y, Abergel RJ, et al (2006) The pathogen-associated iroA gene cluster mediates bacterial evasion of lipocalin 2. Proceedings of the National Academy of Sciences of the United States of America 103: 16502–16507. 17060628 10.1073/pnas.0604636103 PMC1637611

[16] Hantke K, Nicholson G, Rabsch W, Winkelmann G (2003) Salmochelins, siderophores of Salmonella enterica and uropathogenic Escherichia coli strains, are recognized by the outer membrane receptor IroN. Proceedings of the National Academy of Sciences of the United States of America 100: 3677–3682. 12655053 10.1073/pnas.0737682100 PMC152981

[17] Konturek PC, Kania J, Kukharsky V, Raithel M, Ocker M, et al (2004) Implication of peroxisome proliferator-activated receptor gamma and proinflammatory cytokines in gastric carcinogenesis: link to Helicobacter pylori-infection. Journal of pharmacological sciences 96: 134–143. 15492468 10.1254/jphs.fpj04016x

[18] Mahajan S, Dkhar HK, Chandra V, Dave S, Nanduri R, et al (2012) Mycobacterium tuberculosis modulates macrophage lipid-sensing nuclear receptors PPARgamma and TR4 for survival. Journal of immunology 188: 5593–5603. 10.4049/jimmunol.1103038 22544925

[19] Kundu P, De R, Pal I, Mukhopadhyay AK, Saha DR, et al (2011) Curcumin alleviates matrix metalloproteinase-3 and -9 activities during eradication of Helicobacter pylori infection in cultured cells and mice. PloS one 6: e16306. 21283694 10.1371/journal.pone.0016306 PMC3025008

[20] Adachi M, Kurotani R, Morimura K, Shah Y, Sanford M, et al (2006) Peroxisome proliferator activated receptor gamma in colonic epithelial cells protects against experimental inflammatory bowel disease. Gut 55: 1104–1113. 16547072 10.1136/gut.2005.081745 PMC1513267

[21] Dreyer C, Krey G, Keller H, Givel F, Helftenbein G, et al (1992) Control of the peroxisomal beta-oxidation pathway by a novel family of nuclear hormone receptors. Cell 68: 879–887. 1312391 10.1016/0092-8674(92)90031-7

[22] Mansen A, Guardiola-Diaz H, Rafter J, Branting C, Gustafsson JA (1996) Expression of the peroxisome proliferator-activated receptor (PPAR) in the mouse colonic mucosa. Biochemical and biophysical research communications 222: 844–851. 8651933 10.1006/bbrc.1996.0832

[23] Ricote M, Li AC, Willson TM, Kelly CJ, Glass CK (1998) The peroxisome proliferator-activated receptor-gamma is a negative regulator of macrophage activation. Nature 391: 79–82. 9422508 10.1038/34178

[24] Wahli W, Michalik L (2012) PPARs at the crossroads of lipid signaling and inflammation. Trends in endocrinology and metabolism: TEM 23: 351–363. 22704720 10.1016/j.tem.2012.05.001

[25] Su CG, Wen X, Bailey ST, Jiang W, Rangwala SM, et al (1999) A novel therapy for colitis utilizing PPAR-gamma ligands to inhibit the epithelial inflammatory response. The Journal of clinical investigation 104: 383–389. 10449430 10.1172/JCI7145 PMC408529

[26] Yang XY, Wang LH, Chen T, Hodge DR, Resau JH, et al (2000) Activation of human T lymphocytes is inhibited by peroxisome proliferator-activated receptor gamma (PPARgamma) agonists. PPARgamma co-association with transcription factor NFAT. The Journal of biological chemistry 275: 4541–4544. 10671476 10.1074/jbc.275.7.4541

[27] Bassaganya-Riera J, Reynolds K, Martino-Catt S, Cui Y, Hennighausen L, et al (2004) Activation of PPAR gamma and delta by conjugated linoleic acid mediates protection from experimental inflammatory bowel disease. Gastroenterology 127: 777–791. 15362034 10.1053/j.gastro.2004.06.049

[28] Desreumaux P, Dubuquoy L, Nutten S, Peuchmaur M, Englaro W, et al (2001) Attenuation of colon inflammation through activators of the retinoid X receptor (RXR)/peroxisome proliferator-activated receptor gamma (PPARgamma) heterodimer. A basis for new therapeutic strategies. The Journal of experimental medicine 193: 827–838. 11283155 10.1084/jem.193.7.827 PMC2193371

[29] Dubuquoy L, Dharancy S, Nutten S, Pettersson S, Auwerx J, et al (2002) Role of peroxisome proliferator-activated receptor gamma and retinoid X receptor heterodimer in hepatogastroenterological diseases. Lancet 360: 1410–1418. 12424006 10.1016/S0140-6736(02)11395-X

[30] Katayama K, Wada K, Nakajima A, Mizuguchi H, Hayakawa T, et al (2003) A novel PPAR gamma gene therapy to control inflammation associated with inflammatory bowel disease in a murine model. Gastroenterology 124: 1315–1324. 12730872 10.1016/s0016-5085(03)00262-2

[31] Lytle C, Tod TJ, Vo KT, Lee JW, Atkinson RD, et al (2005) The peroxisome proliferator-activated receptor gamma ligand rosiglitazone delays the onset of inflammatory bowel disease in mice with interleukin 10 deficiency. Inflammatory bowel diseases 11: 231–243. 15735429 10.1097/01.mib.0000160805.46235.eb

[32] Nakajima A, Wada K, Miki H, Kubota N, Nakajima N, et al (2001) Endogenous PPAR gamma mediates anti-inflammatory activity in murine ischemia-reperfusion injury. Gastroenterology 120: 460–469. 11159886 10.1053/gast.2001.21191

[33] Saubermann LJ, Nakajima A, Wada K, Zhao S, Terauchi Y, et al (2002) Peroxisome proliferator-activated receptor gamma agonist ligands stimulate a Th2 cytokine response and prevent acute colitis. Inflammatory bowel diseases 8: 330–339. 12479648 10.1097/00054725-200209000-00004

[34] Necela BM, Su W, Thompson EA (2008) Toll-like receptor 4 mediates cross-talk between peroxisome proliferator-activated receptor gamma and nuclear factor-kappaB in macrophages. Immunology 125: 344–358. 18422969 10.1111/j.1365-2567.2008.02849.x PMC2669138

[35] Are A, Aronsson L, Wang S, Greicius G, Lee YK, et al (2008) Enterococcus faecalis from newborn babies regulate endogenous PPARgamma activity and IL-10 levels in colonic epithelial cells. Proceedings of the National Academy of Sciences of the United States of America 105: 1943–1948. 18234854 10.1073/pnas.0711734105 PMC2538862

[36] Kelly D, Campbell JI, King TP, Grant G, Jansson EA, et al (2004) Commensal anaerobic gut bacteria attenuate inflammation by regulating nuclear-cytoplasmic shuttling of PPAR-gamma and RelA. Nature immunology 5: 104–112. 14691478 10.1038/ni1018

[37] Zheng Y, Valdez PA, Danilenko DM, Hu Y, Sa SM, et al (2008) Interleukin-22 mediates early host defense against attaching and effacing bacterial pathogens. Nature medicine 14: 282–289. 10.1038/nm1720 18264109

[38] Berger T, Cheung CC, Elia AJ, Mak TW (2010) Disruption of the Lcn2 gene in mice suppresses primary mammary tumor formation but does not decrease lung metastasis. Proceedings of the National Academy of Sciences of the United States of America 107: 2995–3000. 20133630 10.1073/pnas.1000101107 PMC2840296

[39] Leng X, Ding T, Lin H, Wang Y, Hu L, et al (2009) Inhibition of lipocalin 2 impairs breast tumorigenesis and metastasis. Cancer research 69: 8579–8584. 19887608 10.1158/0008-5472.CAN-09-1934

[40] Dubuquoy L, Jansson EA, Deeb S, Rakotobe S, Karoui M, et al (2003) Impaired expression of peroxisome proliferator-activated receptor gamma in ulcerative colitis. Gastroenterology 124: 1265–1276. 12730867 10.1016/s0016-5085(03)00271-3

[41] Florin L, Hummerich L, Dittrich BT, Kokocinski F, Wrobel G, et al (2004) Identification of novel AP-1 target genes in fibroblasts regulated during cutaneous wound healing. Oncogene 23: 7005–7017. 15273721 10.1038/sj.onc.1207938

[42] Li C, Chan YR (2011) Lipocalin 2 regulation and its complex role in inflammation and cancer. Cytokine 56: 435–441. 21855366 10.1016/j.cyto.2011.07.021

[43] Kundu P, Mukhopadhyay AK, Patra R, Banerjee A, Berg DE, et al (2006) Cag pathogenicity island-independent up-regulation of matrix metalloproteinases-9 and -2 secretion and expression in mice by Helicobacter pylori infection. The Journal of biological chemistry 281: 34651–34662. 16966323 10.1074/jbc.M604574200

[44] Castaneda FE, Walia B, Vijay-Kumar M, Patel NR, Roser S, et al (2005) Targeted deletion of metalloproteinase 9 attenuates experimental colitis in mice: central role of epithelial-derived MMP. Gastroenterology 129: 1991–2008. 16344067 10.1053/j.gastro.2005.09.017

[45] Garg P, Vijay-Kumar M, Wang L, Gewirtz AT, Merlin D, et al (2009) Matrix metalloproteinase-9-mediated tissue injury overrides the protective effect of matrix metalloproteinase-2 during colitis. American journal of physiology Gastrointestinal and liver physiology 296: G175–184. 19171847 10.1152/ajpgi.90454.2008 PMC2643910

[46] Brinckerhoff CE, Matrisian LM (2002) Matrix metalloproteinases: a tail of a frog that became a prince. Nature reviews Molecular cell biology 3: 207–214. 11994741 10.1038/nrm763

[47] Hoiseth SK, Stocker BA (1981) Aromatic-dependent Salmonella typhimurium are non-virulent and effective as live vaccines. Nature 291: 238–239. 7015147 10.1038/291238a0

[48] Imai T, Takakuwa R, Marchand S, Dentz E, Bornert JM, et al (2004) Peroxisome proliferator-activated receptor gamma is required in mature white and brown adipocytes for their survival in the mouse. Proceedings of the National Academy of Sciences of the United States of America 101: 4543–4547. 15070754 10.1073/pnas.0400356101 PMC384783

[49] Wlodarska M, Willing B, Keeney KM, Menendez A, Bergstrom KS, et al (2011) Antibiotic treatment alters the colonic mucus layer and predisposes the host to exacerbated Citrobacter rodentium-induced colitis. Infection and Immunity 79: 1536–1545. 21321077 10.1128/IAI.01104-10 PMC3067531

[50] Schreiber E, Matthias P, Muller MM, Schaffner W (1989) Rapid detection of octamer binding proteins with ‘mini-extracts’, prepared from a small number of cells. Nucleic acids research 17: 6419. 2771659 10.1093/nar/17.15.6419 PMC318318

