# Supplementary figures and images for: Absence of Intestinal PPARγ Aggravates Acute Infectious Colitis in Mice through a Lipocalin-2–Dependent Pathway
Source: PLoS Pathog. 2014 Jan 23;10(1):e1003887. doi: 10.1371/journal.ppat.1003887 (PMC3900641; doi:10.1371/journal.ppat.1003887)

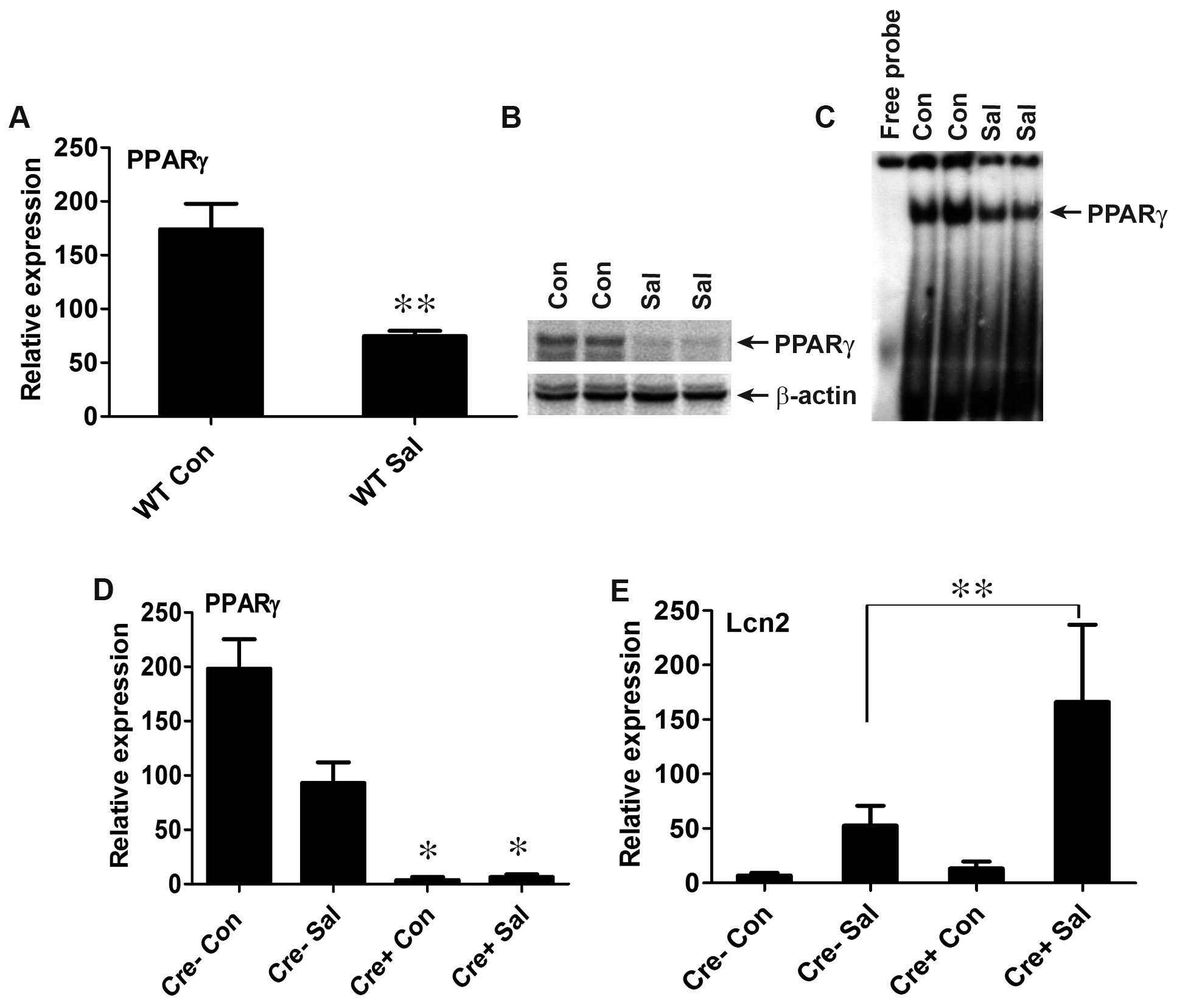

Supplement: Figure S1 — S. Typhimurium down-regulates PPARγ in the cecum during colitis. (A, B, and C) Groups of 8–10-week-old, streptomycin-pretreated C57BL/6 mice (WT) were mock (Con)- or S. Typhimurium (SaI)-infected and sacrificed after 24 h (10 mice per group). PPARγ expression in the cecum was analyzed by real-time PCR (A) and by immunoblotting (B). (C) Electromobility shift assay of PPARγ activity in nuclear extracts from the cecum. (D and E) Groups of age-matched, streptomycin-pretreated PPARγVillinCre+ (Cre+) or littermate control PPARγVillinCre− (Cre−) mice were mock- or S. Typhimurium-infected and sacrificed after 24 h (6–8 mice per group). PPARγ (D) and Lcn2 (E) expression in the cecum was analyzed by real-time PCR. Error bars = ± standard error of the mean. *p<0.005, **p<0.05. (TIF) [file ppat.1003887.s001.tif]

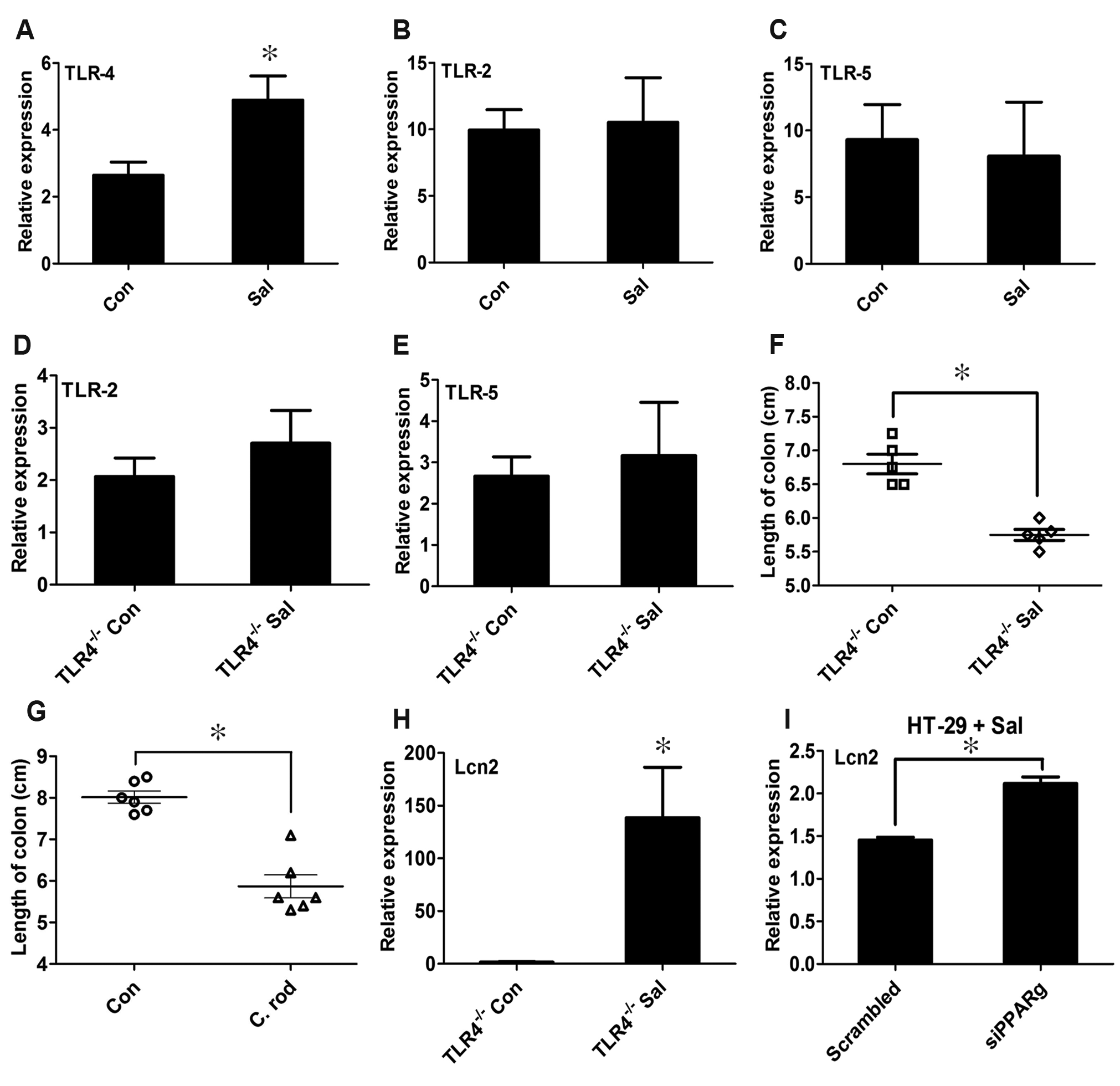

Supplement: Figure S2 — S. Typhimurium down-regulates PPARγ independent of TLR-4 signaling. (A, B, and C) Groups of 8–10-week-old, streptomycin-pretreated C57BL/6 mice were mock (Con)- or S. Typhimurium (SaI)-infected and sacrificed after 24 h (10 mice per group). The expression of TLR-4 (A), TLR-2 (B), and TLR-5 (C) in the colon was analyzed by real-time PCR. (D–F and H) Age-matched, streptomycin-pretreated TLR4−/− mice were mock- or S. Typhimurium-infected and sacrificed after 24 h (5 mice per group). Expression of TLR-4 (D), TLR-2 (E), and Lcn2 (H) in the colon was analyzed by real-time PCR. (F) Quantitation of colon lengths in the respective mouse groups. (G) Metronidazole-pretreated C57BL/6 mice were mock- or C. rodentium-infected, sacrificed 6 days after infection and colon lengths in the respective mice were quantified. (I) HT-29 cells were treated with siRNA directed against PPARγ, infected with S. Typhimurium and the expression of Lcn2 in infected cells was analyzed by real-time PCR. Error bars = ± standard error of the mean. *p<0.005. (TIF) [file ppat.1003887.s002.tif]

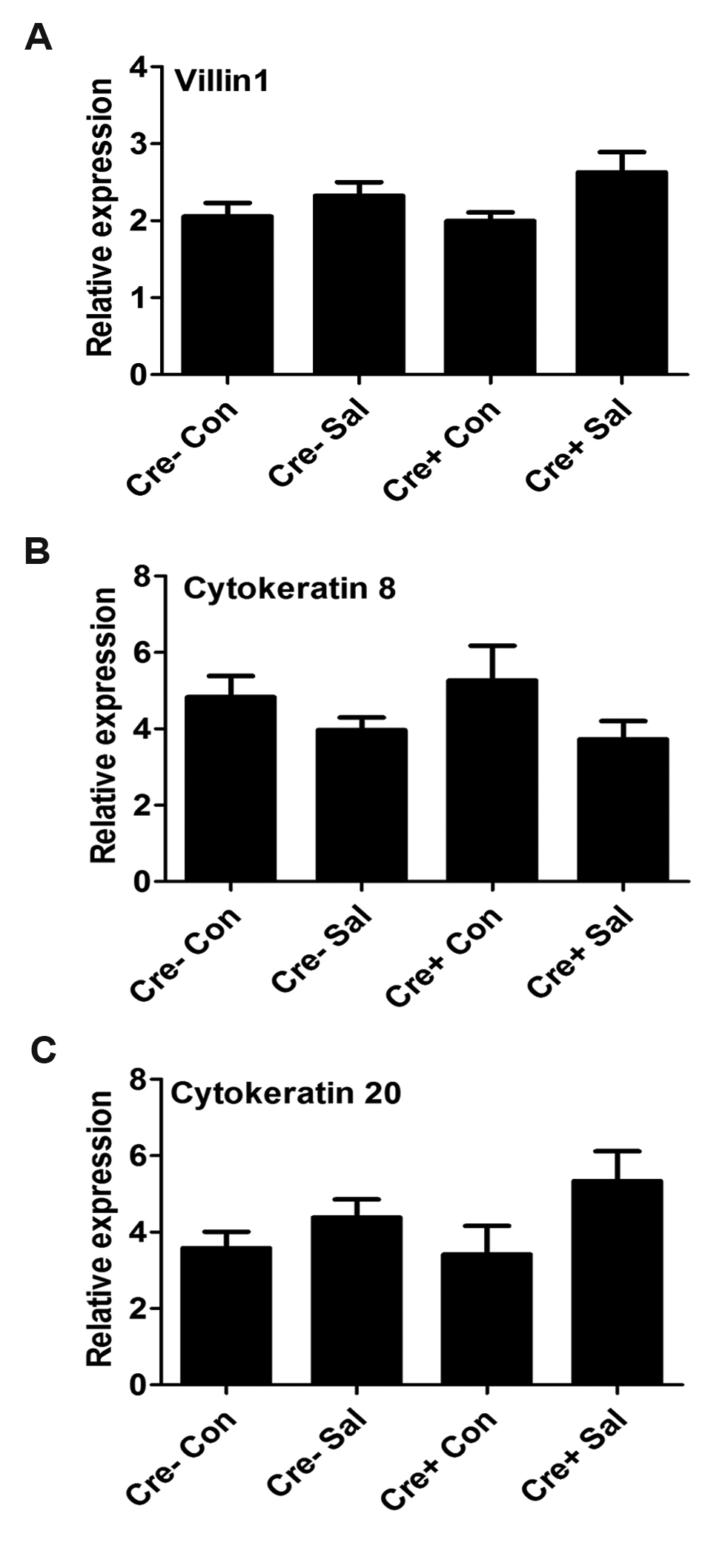

Supplement: Figure S3 — Analysis of epithelial cell markers in colonic scrapings. Groups of age-matched, streptomycin-pretreated PPARγVillinCre+ (Cre+) or littermate control PPARγVillinCre− (Cre−) mice were mock (Con)- or S. Typhimurium (SaI)-infected and sacrificed after 24 h (6–8 mice per group). The expression levels of villin 1 (A), cytokeratin 8 (B), and cytokeratin 20 (C) in colonic scrapings were analyzed by real-time PCR. Error bars = ± standard error of the mean. (TIF) [file ppat.1003887.s003.tif]

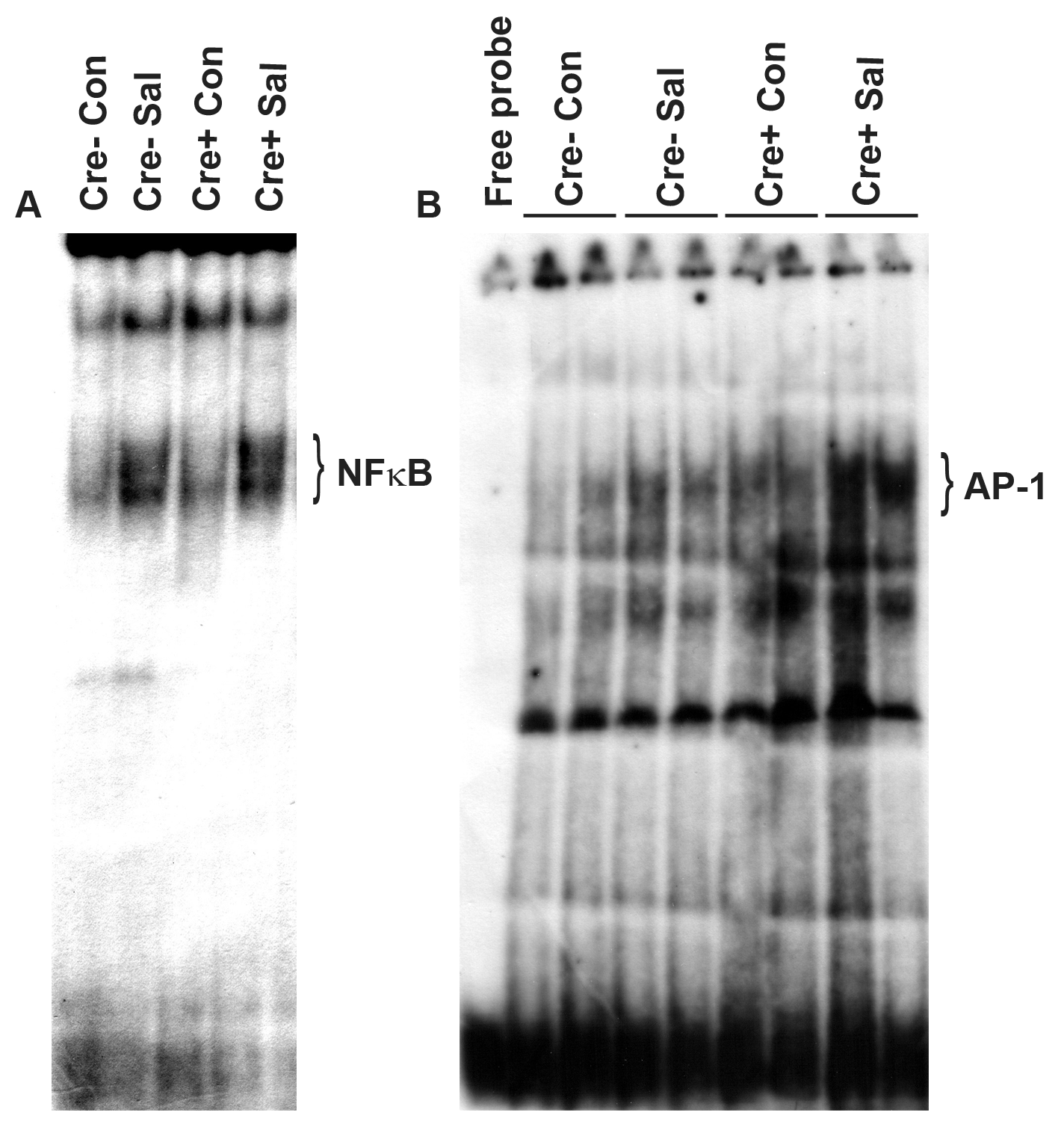

Supplement: Figure S4 — NFκB and AP1 activity in the colons of PPARγVillinCre+ mice after S. Typhimurium infection. Electromobility shift assay of NFκB activity (A) and AP-1 activity (B) in nuclear extracts from colonic scrapings of PPARγVillinCre+ (Cre+) or PPARγVillinCre− (Cre−) mice 24 h after mock (Con)- or S. Typhimurium (SaI)-infection (6 mice per group). (TIF) [file ppat.1003887.s004.tif]

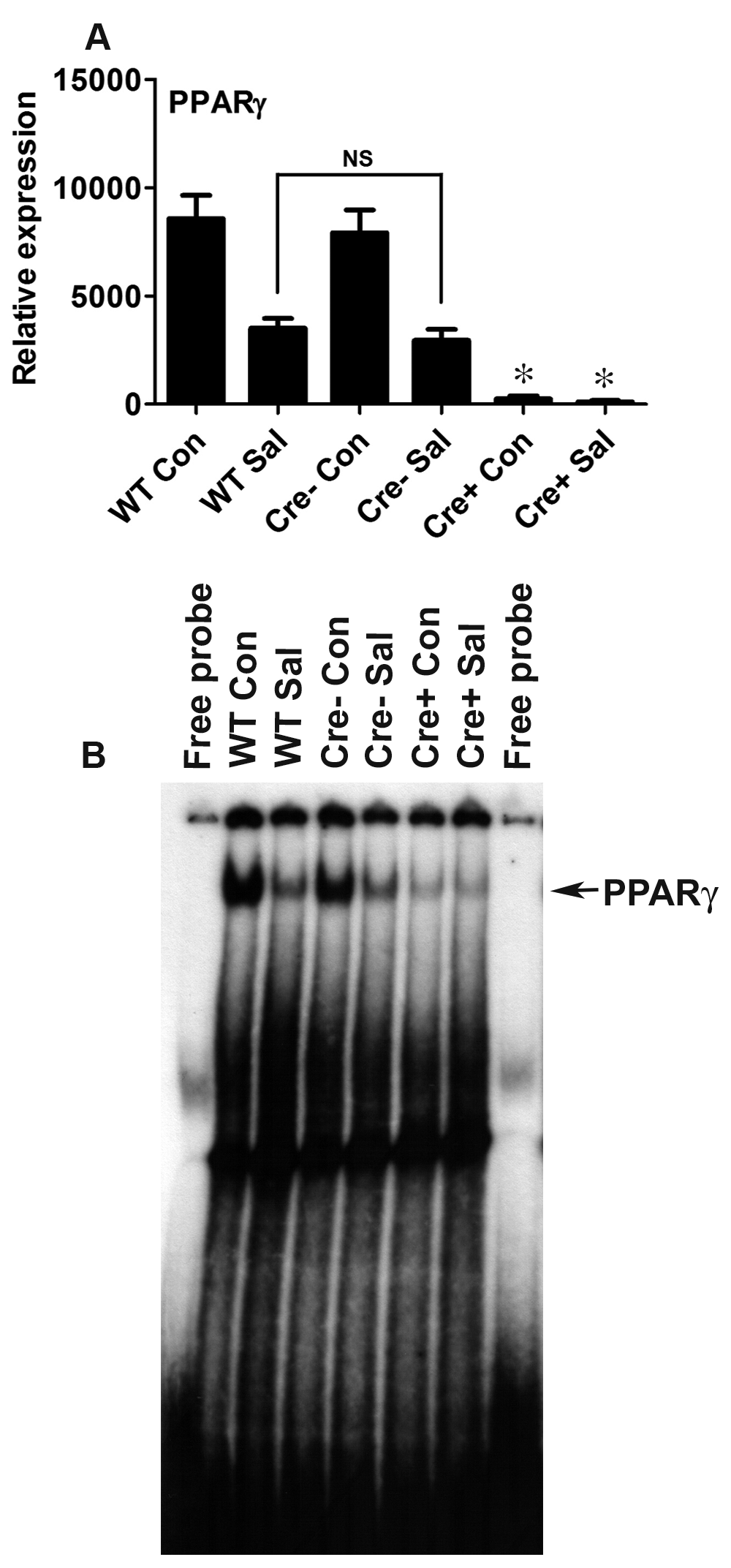

Supplement: Figure S5 — Determination of the efficiency of tissue-specific PPARγ ablation. Groups of 8–10-week-old, streptomycin-pretreated C57BL/6 (WT), PPARγVillinCre+ (Cre+), or littermate control PPARγVillinCre− (Cre−) mice were mock (Con)- or S. Typhimurium (SaI)-infected and sacrificed after 24 h (6–8 mice per group). (A) PPARγ expression in colonic scrapings was analyzed by real-time PCR. Error bars = ± standard error of the mean. *p<0.001 vs. WT or Cre- mice. NS, not significant. (B) Electromobility shift assay of PPARγ activity in nuclear extracts of colonic scrapings. (TIF) [file ppat.1003887.s005.tif]

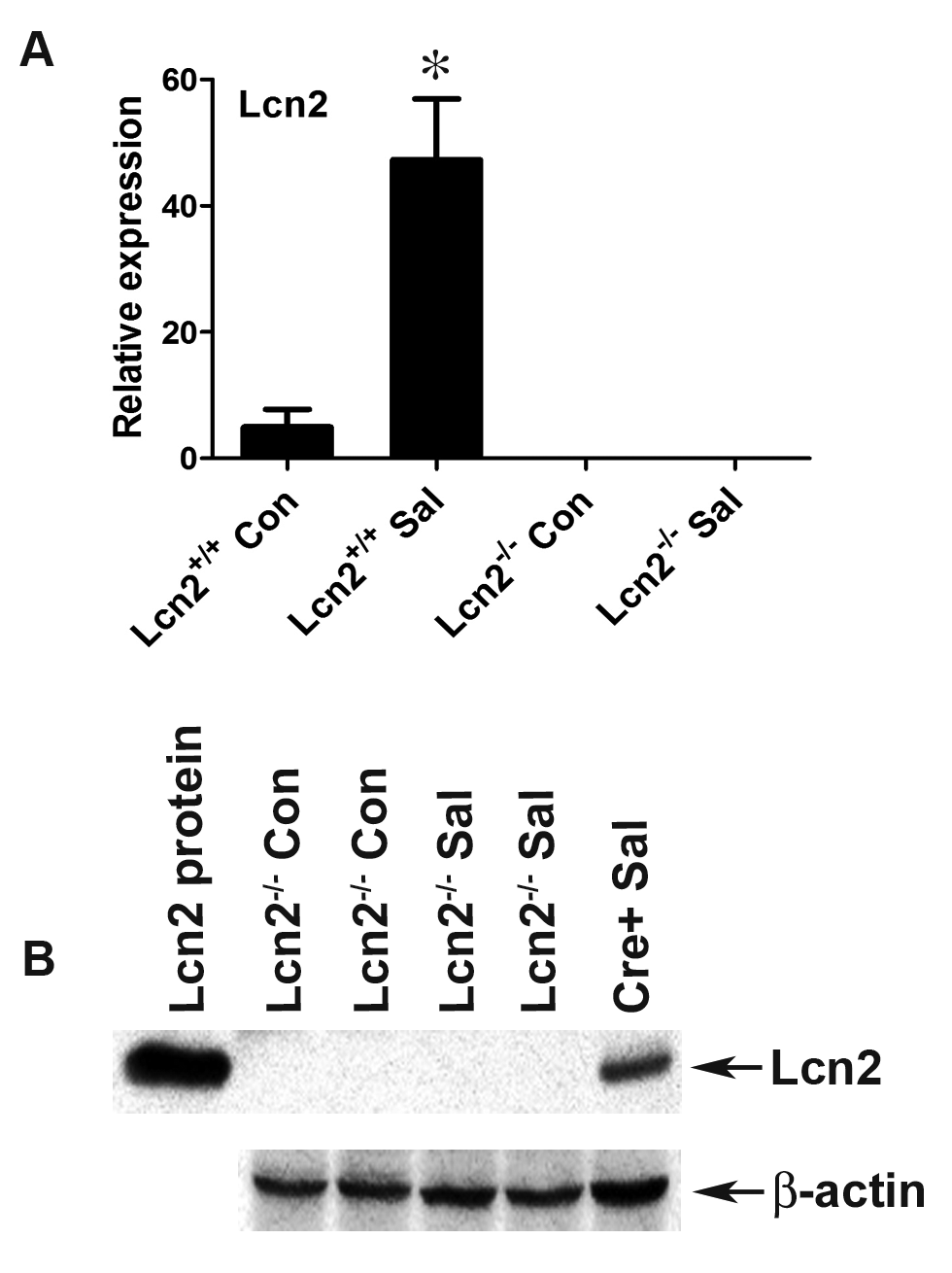

Supplement: Figure S6 — Determination of the efficiency of the Lcn2−/− mouse model. (A and B) Groups of 8–10-week old, streptomycin-pretreated Lcn2+/+ and Lcn2−/− mice were mock (Con)- or S. Typhimurium (SaI)-infected and sacrificed after 24 h (6–8 mice per group). Lcn2 expression in the colon was analyzed by real-time PCR (A) or by immunoblotting (B). Error bars = ± standard error of the mean. *p<0.005 vs. Lcn2+/+ control. (TIF) [file ppat.1003887.s006.tif]

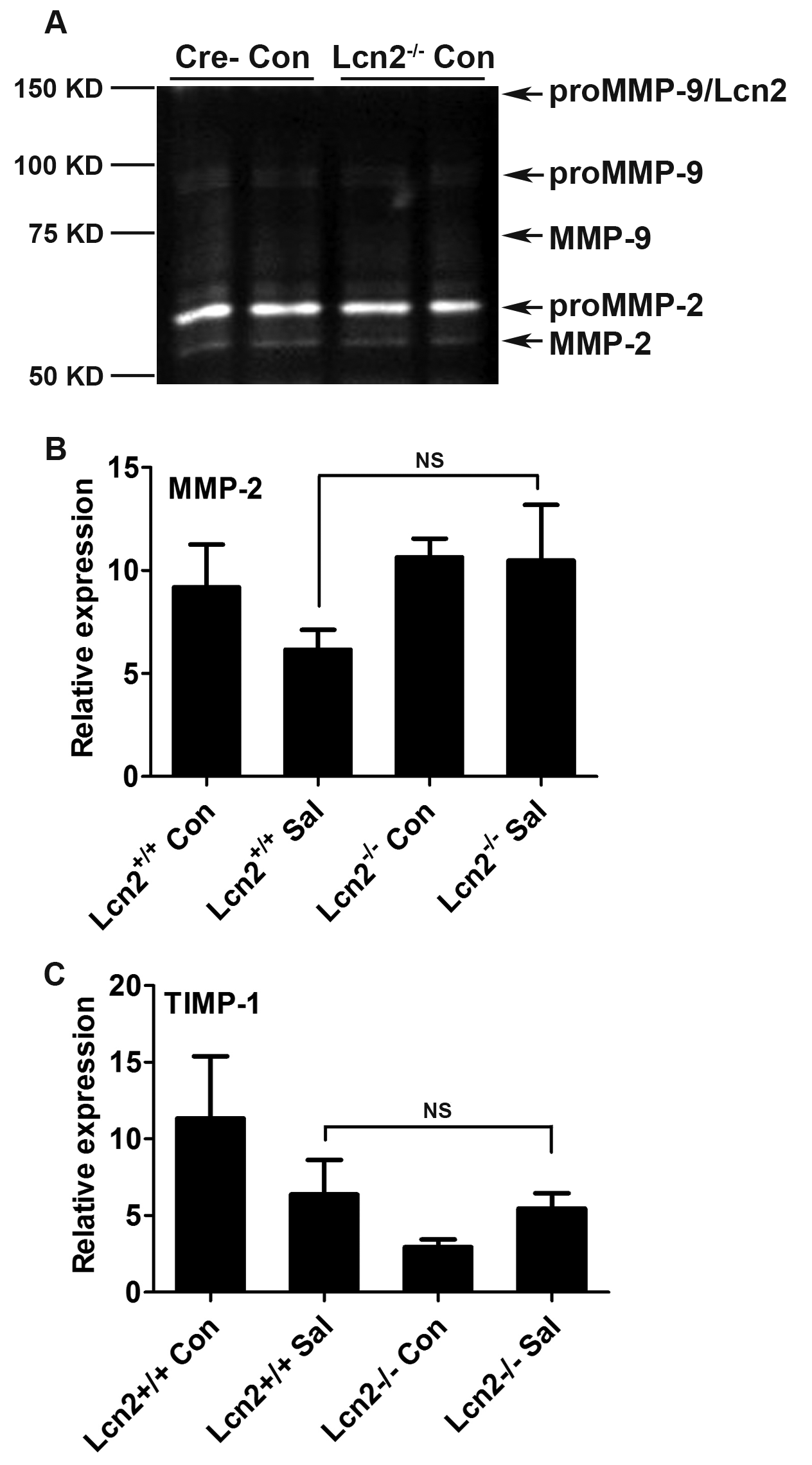

Supplement: Figure S7 — Determination of basal secretion of gelatinases and expression of MMP-2 and TIMP-1 in Lcn2−/− mice. (A) Secretion of MMP-9 and MMP-2 in the colons of mock (Con)-infected PPARγVillinCre− or Lcn2−/− mice (6–8 mice per group) was analyzed by gelatin zymography using gelatin-agarose-purified PBS extracts. Expression levels of MMP-2 (B) and TIMP-1 (C) in the colons of mock- or S. Typhimurium (SaI)-infected Lcn2+/+ and Lcn2−/− mice were measured by real-time PCR (6–8 mice per group). Error bars = ± standard error of the mean. NS, not significant. (TIF) [file ppat.1003887.s007.tif]

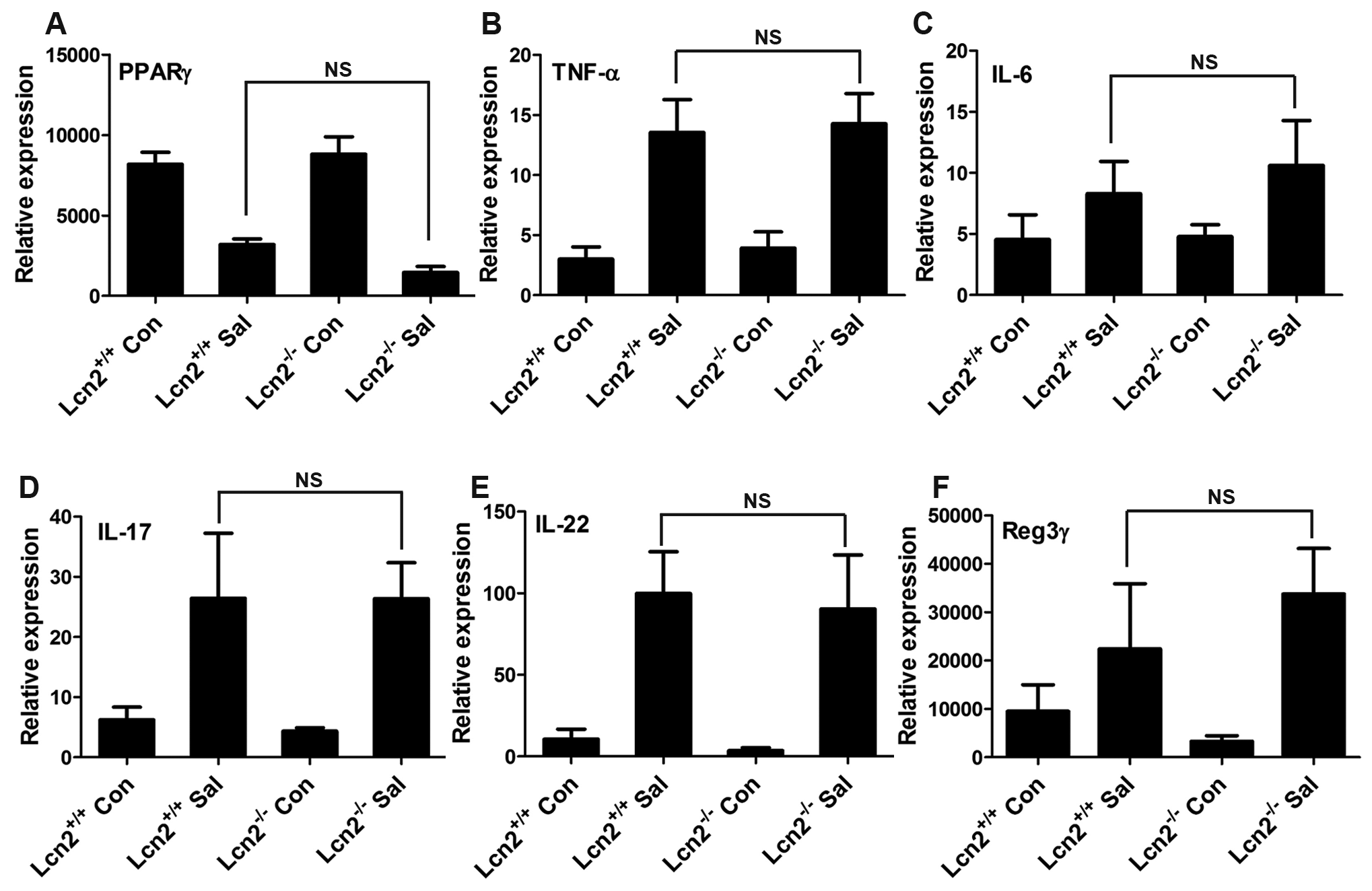

Supplement: Figure S8 — Assessment of mechanistic differences between Lcn2+/+ and Lcn2−/− mice during S. Typhimurium infection. Expression levels of PPARγ (A), TNF-α (B), IL-6 (C), IL-17 (D), IL-22 (E), and Reg3γ (F) in the colons of mock (Con)- or S. Typhimurium (SaI)-infected Lcn2+/+ and Lcn2−/− mice were measured by real-time PCR (6–8 mice per group). Error bars = ± standard error of the mean. NS, not significant. (TIF) [file ppat.1003887.s008.tif]

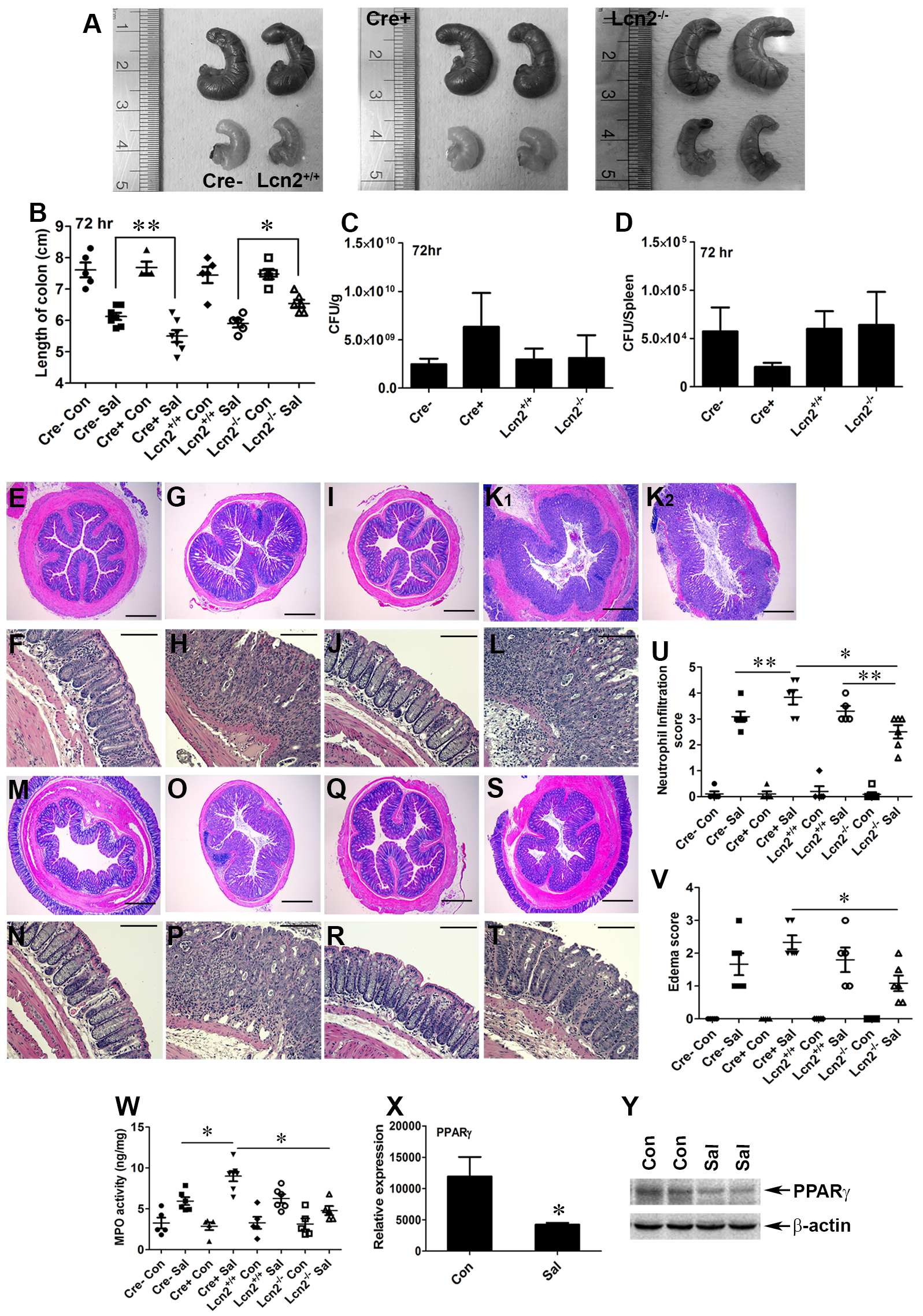

Supplement: Figure S9 — Severity of colitis 72 h after S. Typhimurium infection in mice. Groups of age-matched, streptomycin-pretreated PPARγVillinCre+ (Cre+), littermate control PPARγVillinCre− (Cre−), Lcn2−/−, and littermate control Lcn2+/+ mice were mock (Con)- or S. Typhimurium (SaI)-infected and sacrificed after 72 h (6 mice per group). (A) Macroscopic image of whole cecum after mock or S. Typhimurium infection. (B) Quantitation of colon lengths. Recovery of S. Typhimurium from cecum tissue (C) and spleen (D) 72 h after infection. Sections of colon from these mice were stained with hematoxylin and eosin (E–T). PPARγVillinCre− mice after mock infection (E and F), or after S. Typhimurium infection (G and H). PPARγVillinCre+ mice after mock infection (I and J), or after infection with S. Typhimurium (K and L). Lcn2+/+ mice after mock infection (M and N), or after infection with S. Typhimurium (O and P). Lcn2−/− mice after mock infection (Q and R), or after S. Typhimurium infection (S and T). All scale bars are 500 µm. Pathology scoring was carried out for neutrophil infiltration (U) and edema (V). (W) Myeloperoxidase (MPO) activity in colonic extracts from mice, measured per mg of total protein. PPARγ expression in colonic scrapings from PPARγVillinCre− mice analyzed by real-time PCR (X), and immunoblotting (Y). Error bars = ± standard error of the mean. *p<0.005, **p<0.05 vs. appropriate control or as indicated. (TIF) [file ppat.1003887.s009.tif]
