# Supplementary material for: Absence of Intestinal PPARγ Aggravates Acute Infectious Colitis in Mice through a Lipocalin-2–Dependent Pathway
Source: PLoS Pathog. 2014 Jan 23;10(1):e1003887. doi: 10.1371/journal.ppat.1003887 (PMC3900641; doi:10.1371/journal.ppat.1003887)
Supplement: Table S1 — Details of primers used for real-time RT-PCR analysis of mouse colonic tissues and human cultured cells. (A) The details of the mRNA of interest, sequence of primer pairs with amplicon size used for RT-PCR analysis of mouse colonic tissues. (B) The details of the mRNA of interest, sequence of primer pairs with amplicon size used for RT-PCR analysis of human cultured cells. (DOCX) [file ppat.1003887.s010.docx]

**Table S1.**

**A**

**Details of primers used for real-time RT-PCR analysis of mouse colonic tissues.**

mRNA Primer pairs Sequence Amplicon

size (bp)

*PPARγ*  PPARγ-F 5'-GGTGAACCACTGATATTCAGGA-3' 83

PPARγ-R 5'-AATGGCATCTCTGTGTCAACC-3'

*TNF-α* TNF-α-F 5'-CAAATGGCCTCCCTCTCAT-3' 116

TNF-α-R 5'-CTCCTCCACTTGGTGGTTTG-3'

*IL-6*  IL-6-F 5'-CAAAGCCAGAGTCCTTCAGA -3' 107

IL-6-R 5'-GAGCATTGGAAATTGGGGTA-3'

*IL-17*  IL-17-F 5'-TTTAACTCCCTTGGCGCAAAA-3' 165

IL-17-R 5'-CTTTCCCTCCGCATTGACAC-3'

*IL-22*  IL-22-F 5'-AGACAGGTTCCAGCCCTACA-3' 104

IL-22-R 5'-TCTGGATGTTCTGGTCGTCA-3'

*Lcn2*  Lcn2-F 5'-AATGTCACCTCCATCCTGGT-3' 79

Lcn2-R 5'-CCCTGGAGCTTGGAACAAAT-3'

*Reg3γ*  Reg3γ-F 5'-TTCCTGTCCTCCATGATCAAAA-3' 101

Reg3γ-R 5'-CATCCACCTCTGTTGGGTTCA-3'

*MMP-9* MMP-9-F 5'-GTATGGCTTCTGCCCTACCC-3' 103

MMP-9-R 5'-AGAGTACTGCTTGCCCAGGA-3'

*MMP-2*  MMP-2-F 5'-ACACTGGGACCTGTCACTCC-3' 117

MMP-2-R 5'-TGTCACTGTCCGCCAAATAA-3'

*TIMP-1*  TIMP-1-F 5'-TCCCCAGAAATCAACGAGAC-3' 137

TIMP-1-R 5'-CCACAGAGGCTTTCCATGAC-3'

*TLR-4*  TLR-4-F 5'-TGTTCTTCTCCTGCCTGACA-3' 105

TLR-4-R 5'-CATCAGGGACTTTGCTGAGTT-3'

*TLR-2*  TLR-2-F 5'-AAGATGCGCTTCCTGAATTTG-3' 71

TLR-2-R 5'-TCCAGCGTCTGAGGAATGC-3'

*TLR-5*  TLR-5-F 5'-CCCAGCTTGGATGAAATATCTGTAA-3' 89

TLR-5-R 5'-CCCAGTCTTTTCTTCTTGAACACTTA-3'

*Villin1*  Villin1-F 5'-CCTCATAGGGGAAAAGCAAC-3' 101

Villin1-R 5'-CCAACAGGACGGCTTGATAC-3'

*Cytokeratin 8 Cytokeratin 8*-F 5'-AGCTGAGGCTGAAACCATGT-3' 120

*Cytokeratin 8*-R 5'-TTGATGTTGCGGTTCATCTC-3'

*Cytokeratin 20 Cytokeratin 20*-F 5'-GCCCTCCTCAAAAAGGAACA -3' 112

*Cytokeratin 20*-R 5'-TGATCTCTCCCAGGTTCAGG-3'

*β-actin*  β-actin-F 5′-CTGTATTCCCCTCCATCGTG-3′ 87

β-actin-R 5′-CCTCGTCACCCACATAGGAG-3 ′

**B**

**Details of primers used for real-time RT-PCR analysis of human cultured cells.**

mRNA Primer pairs Sequence Amplicon

size (bp)

*PPARγ*  PPARγ-F 5'-AAGGCCATTTTCTCAAACGA-3' 97

PPARγ-R 5'-ACGGAGCTGATCCCAAAGTT-3'

*Lcn2*  Lcn2-F 5'-GCCTCCCTGAAAACCACATC-3' 66

Lcn2-R 5'-TGTGCACTCAGCCGTCGAT-3'

*β-actin*  β-actin-F 5′-CCTGGCACCCAGCACAAT-3′ 70

β-actin-R 5′-GCCGATCCACACGGAGTACT-3 ′
